# Fluconazole‐induced actin cytoskeleton remodeling requires phosphatidylinositol 3‐phosphate 5‐kinase in the pathogenic yeast *Candida glabrata*

**DOI:** 10.1111/mmi.14110

**Published:** 2018-10-03

**Authors:** Priyanka Bhakt, Raju Shivarathri, Deepak Kumar Choudhary, Sapan Borah, Rupinder Kaur

**Affiliations:** ^1^ Laboratory of Fungal Pathogenesis Centre for DNA Fingerprinting and Diagnostics Hyderabad Telangana India; ^2^ Graduate Studies Manipal Academy of Higher Education Manipal Karnataka India

## Abstract

Known azole antifungal resistance mechanisms include mitochondrial dysfunction and overexpression of the sterol biosynthetic target enzyme and multidrug efflux pumps. Here, we identify, through a genetic screen, the vacuolar membrane‐resident phosphatidylinositol 3‐phosphate 5‐kinase (CgFab1) to be a novel determinant of azole tolerance. We demonstrate for the first time that fluconazole promotes actin cytoskeleton reorganization in the emerging, inherently less azole‐susceptible fungal pathogen *Candida glabrata*, and genetic or chemical perturbation of actin structures results in intracellular sterol accumulation and azole susceptibility. Further, *CgFAB1* disruption impaired vacuole homeostasis and actin organization, and the F‐actin‐stabilizing compound jasplakinolide rescued azole toxicity in cytoskeleton defective‐mutants including the *Cgfab1Δ* mutant. In vitro assays revealed that the actin depolymerization factor CgCof1 binds to multiple lipids including phosphatidylinositol 3,5‐bisphosphate. Consistently, CgCof1 distribution along with the actin filament‐capping protein CgCap2 was altered upon both *CgFAB1* disruption and fluconazole exposure. Altogether, these data implicate CgFab1 in azole tolerance through actin network remodeling. Finally, we also show that actin polymerization inhibition rendered fluconazole fully and partially fungicidal in azole‐susceptible and azole‐resistant *C. glabrata* clinical isolates, respectively, thereby, underscoring the role of fluconazole‐effectuated actin remodeling in azole resistance.

## Introduction

Candida species, the most common cause of opportunistic mycoses, account for 10% of nosocomial bloodstream infections worldwide (Pfaller et al., 2006; Brown et al., 2012). Globally, *Candida albicans* is the most predominant Candida species while the prevalence of *C. glabrata* varies geographically and ranges from second to fourth (Pfaller et al., 2006, 2011; Brown et al., 2012; Montagna et al., 2014). *C. glabrata* isolates are intrinsically less susceptible to the ergosterol biosynthesis inhibitory azole drugs (Pfaller, et al., 2002; Akins, 2005; Pfaller et al., 2011). Furthermore, an increase in the number of *C. glabrata* clinical isolates that display resistance to new β‐glucan synthase‐inhibiting antifungal agents, echinocandins, has also been observed recently (Akins, 2005; Pfaller et al., 2011; Perlin, 2015).

Echinocandins impede the synthesis of the fungal cell wall structural component, β‐glucan, by non‐competitive inhibition of β‐1,3‐glucan synthase (encoded by *CgFKS1* and *CgFKS2* genes), and lead to cell death (Akins, 2005; Perlin, 2015). Echinocandin resistance, primarily due to mutations in *CgFKS* genes, is most prevalent among *C. glabrata* isolates, and alarmingly, many fluconazole‐resistant *C. glabrata* isolates show co‐resistance to echinocandins (Pfaller et al., 2011; Singh‐Babak et al., 2012; Alexander et al., 2013; Perlin, 2015; Castanheira et al., 2016). Echinocandin resistance is also linked with altered cell wall chitin content (Walker et al., 2008).

Azoles inhibit the cytochrome‐P450‐dependent lanosterol 14‐ɑ‐demethylase (encoded by the *ERG11* gene) enzyme of the ergosterol biosynthetic pathway and are fungistatic (Akins, 2005). Dysfunctional mitochondria and increased azole efflux by multidrug resistance (MDR) ATP‐binding cassette membrane transporters (CgCdr1 and CgCdr2) are common azole resistance mechanisms in *C. glabrata* (Sanglard et al., 1999; Izumikawa et al., 2003; Kaur et al., 2004; Tscherner et al., 2011). The Zn_2_Cys_6_ zinc cluster‐containing transcription factor CgPdr1 transcriptionally activates multidrug transporters upon azole exposure (Tsai et al., 2006; Vermitsky et al., 2006). Defects in the mismatch repair pathway are known to lead to the hyper‐mutable phenotype and emergence of MDR in *C. glabrata* (Healey et al., 2016).

We have previously screened *C. glabrata* mutants for altered susceptibility to fluconazole (Kaur et al., 2004; Borah et al., 2011) which is a widely used azole antifungal owing to its high efficacy and bioavailability (Akins, 2005). Here, we build upon our earlier work and uncovered an essential role for the vacuolar membrane‐located phosphatidylinositol (PI) 3‐phosphate 5‐kinase, CgFab1, in azole tolerance in *C. glabrata*. We demonstrate for the first time that fluconazole induces actin cytoskeletal depolarization in a CgFab1‐dependent manner, in part, through the PI 3,5‐bisphosphate [PI(3,5)P2]‐binding protein, CgCof1. We also show that fluconazole‐effectuated actin reorganization contributed to intracellular sterol accumulation. Altogether, our findings advance current understanding of tolerance mechanisms that enable *C. glabrata* cells to survive azole antifungal stress.

## Results

### A Tn*7* insertion mutant screen identifies multiple genes contributing to azole tolerance in *C. glabrata*

**Figure 1:**
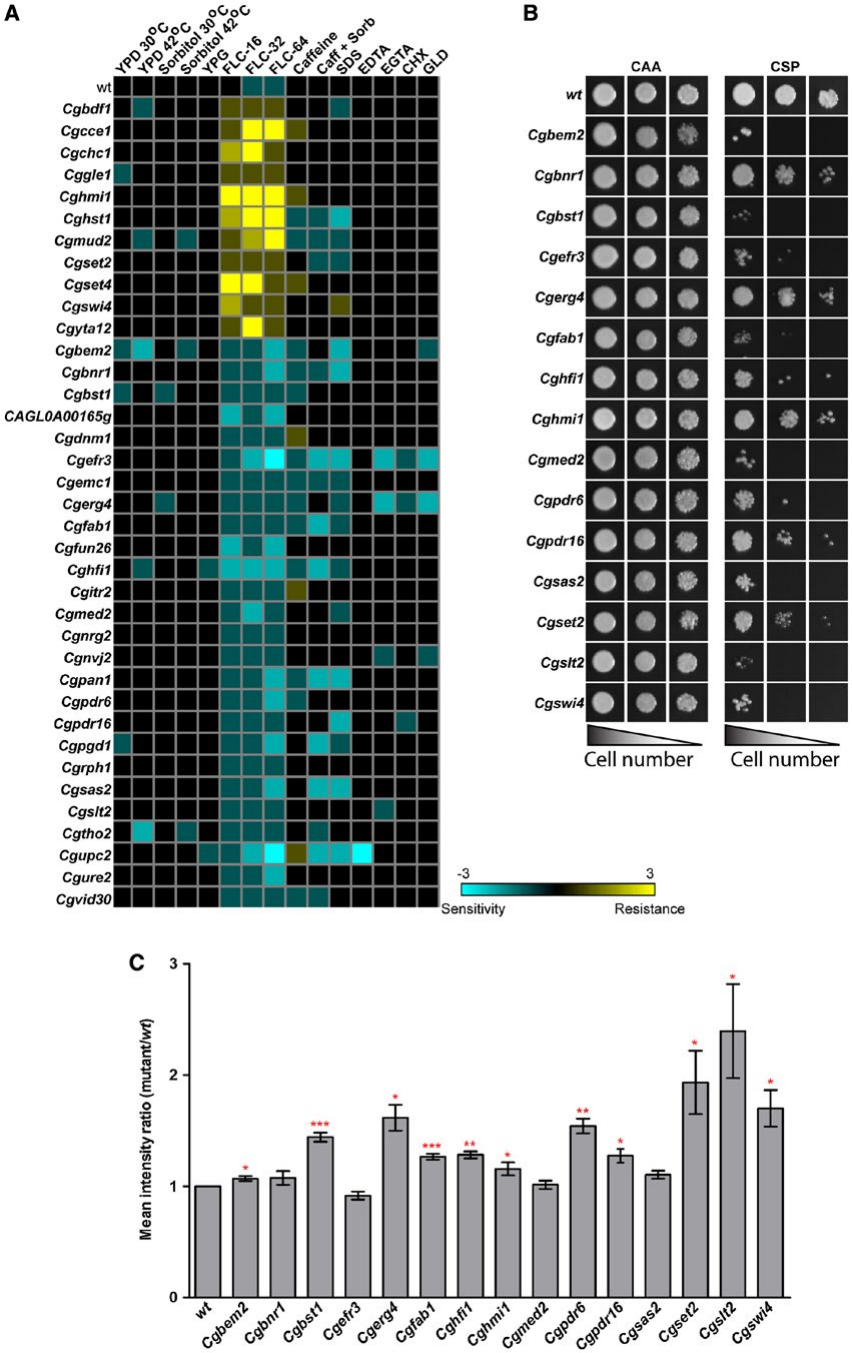
Disruption of several processes leads to increased susceptibility to fluconazole. A. Heat map depicting growth profiles of identified mutants in the presence of diverse stresses. B. CAA liquid‐growth assay‐based assessment of caspofungin (75 ng/ml) sensitivity. Three microliters of 10‐, 100‐ and 1000‐fold diluted cultures were spotted and images were captured after 1 day of growth at 30^°^C. C. Flow cytometry‐based cell wall chitin measurement of caspofungin‐sensitive Tn*7* insertion mutants. Data represent mean ± SEM (n = 3–7). *p < 0.05; ** p < 0.01, ***p < 0.001; paired two‐tailed Student's t‐test. [Colour figure can be viewed at http://www.wileyonlinelibrary.com]

A library of 18,350 random Tn*7* insertion mutants of *C. glabrata* had been screened for altered fluconazole susceptibility in two batches (Kaur et al., 2004; Borah et al., 2011). The first screen of 9,216 mutants revealed that Tn*7* insertions in calcium signaling and RNA polymerase II coactivation genes, and mitochondria function and ribosome biogenesis genes, rendered *C. glabrata* cells more and less susceptible, respectively, to fluconazole (Kaur et al., 2004). Our second plate‐based growth screen of 9,134 mutants identified 74 and 70 mutants to be fluconazole sensitive and resistant respectively (Borah et al., 2011). Further, we screened 74 identified sensitive mutants for viability loss in the presence of fluconazole and found 7 genes to be essential for the survival of fluconazole stress (Borah et al., 2011). Here, we present a detailed analysis of the remaining fluconazole‐sensitive as well as fluconazole‐resistant mutants identified in the second screen (Borah et al., 2011). Tn*7* insertion mapping and sequencing analysis in identified mutants revealed a set of 37 unique genes contributing to azole tolerance with disruption of 26 and 11 genes leading to elevated and diminished susceptibility, respectively, to fluconazole (Fig. 1A, Supporting Information Table S1). Of these, 10 genes have previously been implicated in azole response in *C. glabrata* (Kaur et al., 2004; Borah et al., 2011; Nagi et al., 2011; Tscherner et al., 2011; Orta‐Zavalza et al., 2013) (Supporting Information Table S1). Phenotypic characterization of 37 identified mutants revealed varied susceptibility to other stresses with 15 mutants, with altered fluconazole susceptibility, also exhibiting increased sensitivity to caspofungin (Fig. 1B). Of 15 mutants, 11 displayed increased cell wall chitin content (Fig. 1C), suggesting that altered cell wall composition may partly account for their varied susceptibility to azole and echinocandin antifungals. Overall, our mutant screen data reflect some overlap in mechanisms that contribute to the cellular response to azoles and echinocandins.

### MDR genes are activated in the *Cgfab1Δ* mutant upon fluconazole exposure

**Figure 2:**
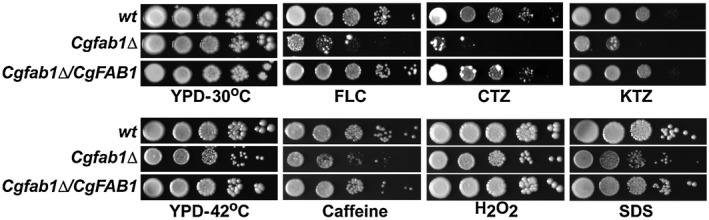
The *Cgfab1Δ* mutant displays sensitivity to azole antifungals. Serial dilution cell spotting analysis. Fluconazole (FLC), clotrimazole (CTZ), ketoconazole (KTZ), SDS, caffeine and hydrogen peroxide (H_2_O_2_) were used at a concentration of 16 µg/ml, 2 µg/ml, 4 µg/ml, 0.05%, 10 mM and 25 mM respectively.

Of 27 newly identified genes, we selected *CgFAB1*, which encodes a putative 1‐phosphatidylinositol‐3‐phosphate 5‐kinase, for further analysis, as it has not been associated with azole tolerance in any fungal pathogen. The *CgFAB1* gene is uncharacterized in *C. glabrata*; however, the product of its ortholog in *Saccharomyces cerevisiae* is the sole vacuolar membrane kinase that generates phosphatidylinositol 3,5‐bisphosphate [PI(3,5)P2] from phosphatidylinositol 3‐phosphate (PI3P), and is involved in vacuolar protein sorting and homeostasis (Cooke et al., 1998; Odorizzi et al., 1998). We created a deletion strain for the *CgFAB1* gene, and found it to be sensitive to azoles (fluconazole, clotrimazole and ketoconazole), and cell membrane (SDS) and cell wall (caffeine) stressors (Fig. 2). The growth of the *Cgfab1Δ* mutant was also slightly impaired in the presence of the oxidative stressor, hydrogen peroxide, and at high temperature (Fig. 2). These results implicate CgFab1 in response to azole, cell membrane and cell wall stress.

Fluconazole exposure leads to overexpression of MDR efflux pumps, and any deficiency in this response leads to drug sensitivity (Tscherner et al., 2011). Hence, we examined transcript levels of genes encoding the multidrug transporter CgCdr1 and the transcription activator of MDR genes CgPdr1 in wild‐type (*wt*) and *Cgfab1Δ* cells. Both strains responded to fluconazole via upregulation of *CgCDR1* and *CgPDR1* genes (Supporting Information Fig. S1A). Consistently, fluconazole‐treated *wt* and *Cgfab1Δ* cells displayed a 1.5‐fold increased efflux of the MDR pump substrate rhodamine 6G (Supporting Information Fig. S1B), thereby precluding any effect of CgFab1 on transcriptional activation and functions of *CgPDR1* and *CgCDR1* genes.

Next, we examined the role of CgFab1 in acquired azole resistance. Gain‐of‐function (GOF) mutations in the *CgPDR1* gene lead to overexpression of MDR efflux pumps, including CgCdr1, resulting in high levels of azole resistance in clinical settings (Ferrari et al., 2009). We expressed the *CgPDR1* hyperactive allele, containing *L280F* mutation in its regulatory domain (Ferrari et al., 2009; Borah et al., 2014), in *Cgfab1Δ* cells, and checked growth in the fluconazole medium. Both *wt* and *Cgfab1Δ* cells carrying GOF CgPdr1 exhibited robust growth in fluconazole at a concentration of 64 µg/ml (Supporting Information Fig. S1C) indicating a non‐requirement for CgFab1 in CgPdr1‐dependent acquired azole resistance. Together, these data suggest that, despite involvement in basal azole tolerance, CgFab1 is dispensable for efflux‐mediated azole resistance.

### CgFab1 is required for vacuole homeostasis

**Figure 3:**
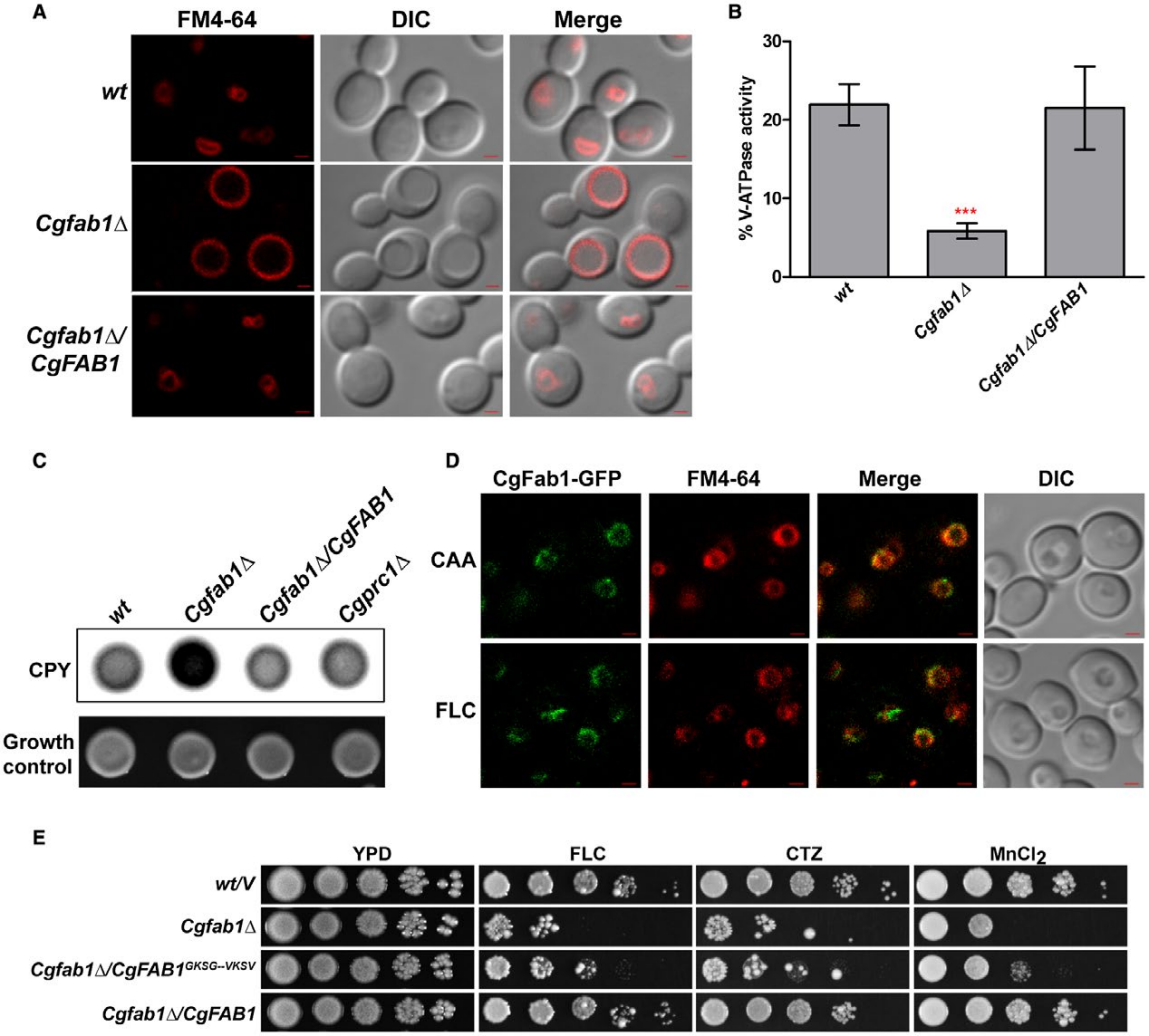
The *Cgfab1Δ* mutant displays perturbed vacuolar functions. A. Representative confocal microscopy images showing the vacuole size in log‐phase *Candida glabrata* cultures. DIC, differential interference contrast; Bar = 1 µm. B. Spectrophotometric measurement of the V‐ATPase activity in purified vacuolar fractions. Data represent the mean ± SEM of at least four independent experiments. ***p < 0.001; unpaired two‐tailed Student’s t‐test. C. Colony‐blot analysis to assess carboxypeptidase Y (CPY) secretion. *C. glabrata* cells were spotted on CAA medium, overlaid with a nitrocellulose membrane and incubated at 30°C for 18 h. The membrane was probed with the polyclonal anti‐CPY antibody. Growth control shows equal growth for all strains. *Cgprc1Δ* is deleted for the CPY‐encoding *CgPRC1* gene, and was used as a negative control. D. Representative confocal images showing localization of CgFab1‐GFP on the FM4‐64 ‐stained vacuolar membrane in 4 h fluconazole (FLC, 16 µg/ml)‐treated and CAA‐grown (CAA) cells. Bar = 1 µm. E. Serial dilution spotting analysis. Fluconazole (16 µg/ml; FLC), clotrimazole (2 µg/ml; CTZ) and manganese chloride (3 mM; MnCl_2_) were used [Colour figure can be viewed at http://www.wileyonlinelibrary.com].

To identify the mechanistic basis underlying azole susceptibility of the *Cgfab1Δ* mutant, we decided to first decipher the role of CgFab1. CgFab1 is a 242 kDa protein with a predicted N‐terminus PI3P‐binding FYVE, a chaperonin‐like and a conserved C‐terminus PI kinase domain (Supporting Information Fig. S2A). Since Fab1 in *S. cerevisiae* is implicated in vacuole homeostasis (Cooke et al., 1998; Odorizzi et al., 1998), we first checked vacuolar morphology in the *Cgfab1Δ* mutant. Staining of log‐phase *Cgfab1Δ* cells with the vacuole membrane‐specific dye FM4‐64 revealed that, compared to 10% *wt* cells, 82% of the mutant population contained large vacuoles (Fig. 3A). Next, we purified vacuolar membranes and measured vacuolar (V) ATPase activity in the *Cgfab1Δ* mutant. Compared to *wt* and complemented strains, the *Cgfab1Δ* mutant displayed 73% reduced V‐ATPase activity (Fig. 3B), which probably results in defective functioning of the vacuole. Consistently, the *Cgfab1Δ* mutant showed increased sensitivity to manganese chloride and zinc chloride (Supporting Information Fig. S2B) reflective of perturbed metal ion storage/homeostasis.

Further, we checked the effect of *CgFAB1* deletion on the delivery of the vacuolar lumenal hydrolase carboxypeptidase Y (CPY), as PI kinases are involved in intracellular trafficking events (Lindmo and Stenmark, 2006; Rai et al., 2015). Under normal conditions, CPY undergoes proteolytic cleavages during its transit, through the Golgi, en route to the vacuole with final enzyme processing occurring in the vacuole (Bairwa et al., 2014). A higher amount of CPY was secreted into the medium in the *Cgfab1Δ* mutant, which indicates CPY mislocalization (Fig. 3C). Consistently, 50% lower intracellular CPY levels in the mutant (Supporting Information Fig. S2C) underscored a role for CgFab1 in sorting of CPY to the vacuole.

For cellular localization studies, we created the CgFab1‐GFP fusion protein, and first verified its functionality via rescue of the *Cgfab1Δ* mutant phenotypes (Supporting Information Fig. S2D). CgFab1‐GFP was found to be localized to the vacuolar membrane under both normal and fluconazole‐treated conditions (Fig. 3D). Together, these data suggest that CgFab1 is a vacuolar membrane protein and regulates vacuole structure and functions.

Our attempts to determine the PI3P‐5 kinase activity of CgFab1 through a thin‐layer chromatography‐based in vitro kinase assay were unsuccessful probably due to reduced expression of the CgFab1 protein. Furthermore, we could not measure cellular PI(3,5)P2 levels in *wt* and *Cgfab1Δ* strains through high‐pressure liquid chromatography due to technical limitations. Keeping this in view, we decided to mutate two glycine residues at positions 1867 and 1870, in the highly conserved ‘GKSG’ sequence in the predicted kinase domain of CgFab1, to valine residues and examined the role of the CgFab1 mutant protein in antifungal tolerance and vacuole functions. Of note, substitutions of these glycine residues in *S. cerevisiae* Fab1 resulted in impaired lipid kinase activity (Gary et al., 1998). Expectedly, the *Cgfab1Δ* mutant carrying CgFab1^G1867/1870V^ was sensitive to both azole and metal ion stress (Fig. 3E), indicating that Gly‐867 and Gly‐1870 are required for the functioning of CgFab1.

Fluconazole has been shown to impair vacuolar acidification, as ergosterol is pivotal to the functioning of the vacuolar H^+^‐ATPase (Zhang et al., 2010). Therefore, we next examined if mutants with defective vacuolar functions (reduced vacuolar ATPase activity and metal ion susceptibility) and large‐sized vacuole display increased sensitivity to azole antifungals. To this end, we checked the growth of *C. glabrata vps15Δ*,* vps34Δ* (Rai et al., 2015) and *yps1‐11Δ* (Bairwa et al., 2014) mutants, which lack the regulatory‐PI3K subunit, catalytic‐PI3K subunit and 11 cell surface‐associated aspartyl proteases, respectively, in the azole‐containing medium. Notably, these mutants have been reported to missort CPY, contain large vacuoles and display sensitivity to several stresses (Bairwa et al., 2014; Rai et al., 2015). The *Cgyps1‐11Δ* mutant has also been reported to display reduced V‐ATPase activity (Bairwa et al., 2014), while the *vps15Δ* and *vps34Δ* mutants in *S. cerevisiae* are known to have lower V‐ATPase activity (Sambade et al., 2005). All three *C. glabrata* mutants, *Cgvps15Δ*,* Cgvps34Δ* and *Cgyps1‐11Δ*, exhibited wild‐type‐like growth in the presence of azole antifungals (Supporting Information Fig. S2E), indicating that the dysfunctional large vacuole per se does not lead to azole susceptibility.

### Fluconazole exposure alters actin dynamics and induces actin depolarization

A *S. cerevisiae* mutant with decreased V‐ATPase activity has been reported to contain abnormal filamentous (F)‐actin distribution (Zhang et al., 1998). As the *Cgfab1Δ* mutant exhibited reduced V‐ATPase activity, we decided to probe the role of CgFab1 in the organization of the actin network. F‐actin in *S. cerevisiae* consists of three dynamic structures: cortical patches, cables and contractile rings (Thevissen et al., 2007). Actin patches, consisting of branched actin filaments, assemble primarily at the bud and the mother‐bud neck, and facilitate endocytosis (Moseley and Goode, 2006). Actin cables, which are polarized linear bundles of actin filaments, traverse along the length of the cell and are pivotal to the transport of organelles and vesicles to the growing bud (Moseley and Goode, 2006; Mishra et al., 2014). Contractile rings, formed by unbranched anti‐parallel actin bundles, are required for cytokinesis (Mishra et al., 2014). Actin cytoskeleton in growing cells is usually polarized with actin patches associated with actively secreting regions and buds, with actin cables oriented in the direction of the patch clusters (Moseley and Goode, 2006; Mishra et al., 2014).

**Figure 4:**
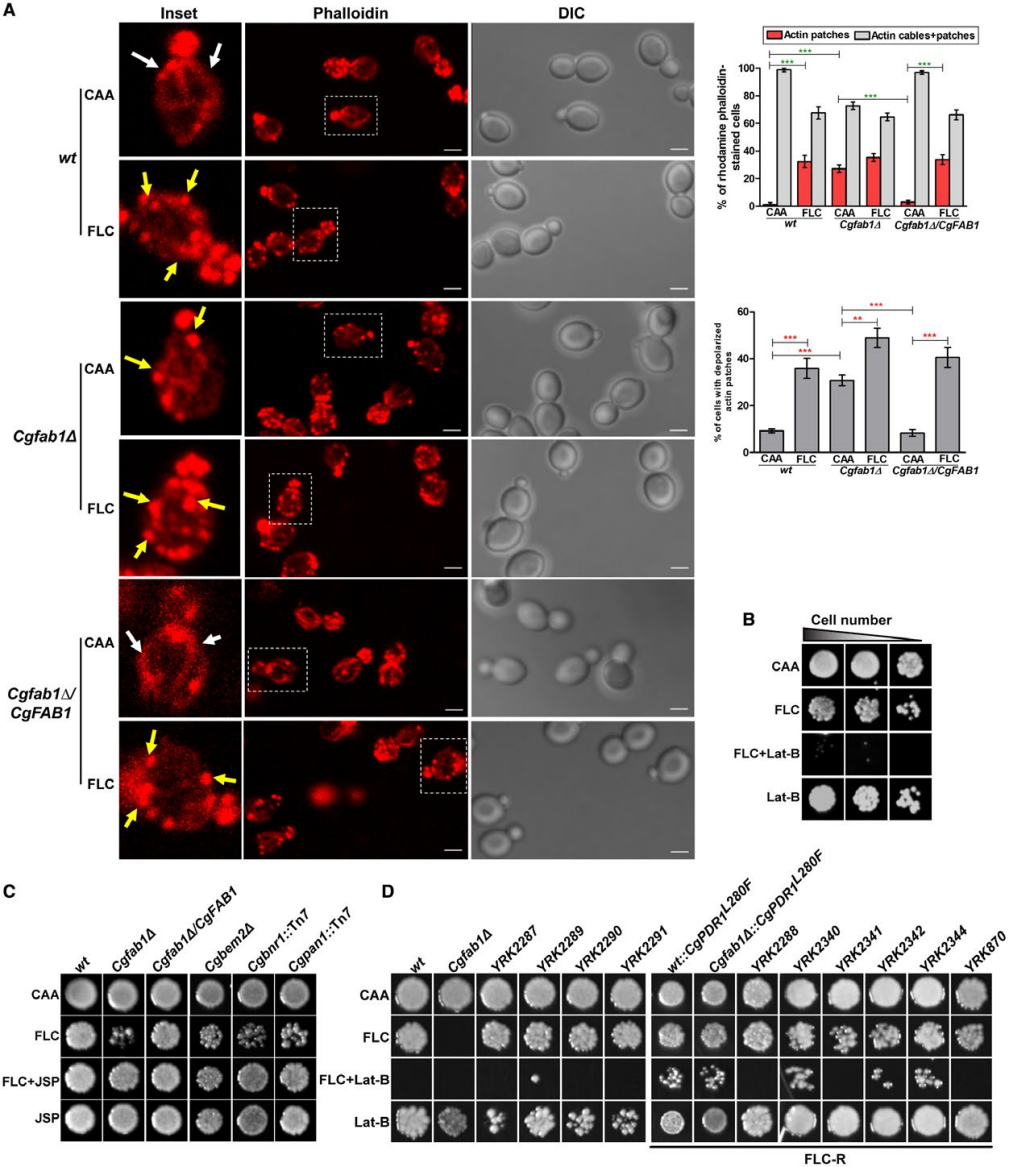
Actin cytoskeleton reorganization is pivotal to azole stress survival. A. Representative maximum‐intensity projection of Z‐stack fluorescence confocal images showing diminished actin cables and depolarized actin patches in azole‐treated cells. White and yellow arrows mark cables and patches respectively. Inset shows zoomed image of the boxed area. For each strain/condition, a minimum of 150 small‐budded cells displaying stained actin were counted. Data (mean ± SEM) are presented as the percentage of cells containing either actin patches exclusively or both actin cables and patches, and the percentage of cells containing depolarized actin patches, on the right side of the image panels. **p < 0.01; ***p < 0.001; Unpaired, two‐tailed, Student's t‐test. Bar = 2 µm. B. Liquid growth assay‐based analysis of the synergistic effect of fluconazole (128 µg/ml; FLC) and latrunculin B (40 µM; Lat‐B). Three microliters of 100‐, 500‐ and 1000‐fold diluted cultures were spotted and images were captured after 2 days of growth at 30°C. C. Liquid growth assay‐based analysis of jasplakinolide (750 ng/ml; JSP)‐mediated rescue of fluconazole (48 µg/ml; FLC) sensitivity. D. Liquid growth assay‐based analysis of the combinatorial action of fluconazole (256 µg/ml) and Lat‐B (40 µM) in clinical isolates. FLC‐R indicates fluconazole‐resistant isolates. [Colour figure can be viewed at http://www.wileyonlinelibrary.com]

To investigate if actin cytoskeleton organization is altered in the *Cgfab1Δ* mutant, we labeled log‐phase mutant cells with rhodamine phalloidin, which stains F‐actin, and counted the number of stained small‐budded cells containing actin cables and/or patches. Compared to *wt*, the percentage of *Cgfab1Δ* cells containing actin cables was lower, and actin cables were shorter in size (Fig. 4A). Further, cortical actin patches were not ordinarily confined to emerging buds, and the percentage of cells with actin patches was about 22‐fold higher (Fig. 4A). Notably, 31% of *Cgfab1Δ* cells displayed actin patch depolarization as compared to 9% of *wt* cells (Fig. 4A). Together, this distinct pattern of actin structures implicates CgFab1 in proper assembly of the actin cytoskeleton in *C. glabrata*.

Next, we examined the effects of fluconazole on actin dynamics in *wt* cells. Fluconazole treatment led to a decline in the percentage of cells containing patches and visible actin cables, and a 33% increase in the number of cells with exclusive actin patches (Fig. 4A). However, these patches were depolarized and randomly distributed in treated cells (Fig. 4A). Compared to 9% untreated cells, actin patch depolarization was observed in 36% of fluconazole‐treated cells (Fig. 4A). These data suggest that fluconazole exposure alters F‐actin structure, and are consistent with an earlier study reporting miconazole‐induced stabilization of the actin cytoskeleton in *S. cerevisiae* (Thevissen et al., 2007). However, contrary to that report, azole‐mediated increase in reactive oxygen species production was not observed in our study (data not shown). Notably, although fluconazole exposure led to no significant change in the cellular distribution of actin cables and patches, the percentage of cells with depolarized actin patches was found to be higher in the *Cgfab1Δ* mutant (Fig. 4A). Importantly, fluconazole‐induced actin network reorganization in the *Cgfab1Δ*‐complemented strain was similar to that of *wt* cells (Fig. 4A). Altogether, these data suggest that fluconazole leads to actin cytoskeletal reorganization, and CgFab1 contributes to this process.

Further, to verify the role of actin remodeling in azole tolerance in *C. glabrata*, we performed three experiments. First, we examined fluconazole toxicity in the presence of latrunculin B (Lat‐B), which inhibits actin polymerization through cell viability measurement. The combined treatment of fluconazole and Lat‐B rendered *wt* cells inviable (Fig. 4B). This synergistic action of fluconazole with the actin polymerization inhibitor suggests that actin cytoskeletal reorganization is required to survive azole stress. Of note, chemogenomic profiling analysis has previously shown cytotoxic synergy between fluconazole and latrunculin‐A in *S. cerevisiae* and *C. albicans* (Jansen et al., 2009). Second, we observed a 2.5‐fold decrease in *CgFAB1* transcript levels upon fluconazole exposure (Supporting Information Fig. S3A). Third, we checked whether the inability to restructure the actin cytoskeleton accounts for azole susceptibility of the *Cgfab1Δ* mutant. In order to achieve this, we treated cells simultaneously with fluconazole and the F‐actin–stabilizing compound, jasplakinolide (Lee et al., 1998). We observed jasplakinolide‐mediated rescue of azole sensitivity in the *Cgfab1Δ* mutant as well as in three other actin cytoskeletal defective mutants, *Cgbnr1*,* Cgpan1* and to a lower extent, *Cgbem2Δ* (Fig. 4C), identified in the mutant screen (Supporting information Table S1). Although, owing to competitive binding of phalloidin and jasplakinolide to actin, we could not check jasplakinolide‐mediated restoration of the actin cytoskeletal defect in the *Cgfab1Δ* mutant, the azole sensitivity rescue is indicative of impaired actin remodeling as a cause of azole toxicity in the mutant. Importantly, jasplakinolide treatment could not complement the large vacuole phenotype of the *Cgfab1Δ* mutant (Supporting Information Fig. S3B), further corroborating that vacuole dysfunctions are unlikely to account for azole sensitivity of the mutant.

Actin structures are dynamic, and F‐actin undergoes continuous cycles of polymerization and depolymerization in actin cables (Moseley and Goode, 2006; Mishra et al., 2014). Our data suggest that fluconazole exposure leads to actin depolarization, as reflected in randomly distributed actin patches and short cables. This depolarization will require disassembly of existing polarized actin structures and reassembly of actin monomers. Conceivably, actin polymerization inhibition will adversely affect the actin filament reassembly, thereby rendering the fluconazole–actin polymerization inhibitor combination cidal. The *Cgfab1Δ* mutant contained fewer actin cables which could be due to unstable actin filaments and/or defective actin assembly. Jasplakinolide is likely to prevent the actin filament disassembly, thereby, circumventing the fluconazole effect and rescuing drug sensitivity of the *Cgfab1Δ* mutant.

### Lat‐B and fluconazole also act synergistically in clinical isolates of *C. glabrata*

To check if impeding actin remodeling renders fluconazole fungicidal in clinical isolates of *C. glabrata*, we determined cell viability in a panel of 10 strains displaying varied fluconazole susceptibility (Supporting Information Fig. S4). We found that both fluconazole and Lat‐B combinations were lethal in four clinical isolates and *Cgfab1Δ* mutant (Fig. 4D). Furthermore, Lat‐B also acted synergistically with fluconazole in six azole‐resistant clinical isolates and hyperactive CgPdr1‐containing laboratory strains, as their growth was impaired in the medium containing both Lat‐B and fluconazole (Fig. 4D). These data highlight the relevance of fluconazole‐induced actin remodeling in clinical isolates.

### Distribution of the actin filament severing and capping protein is altered upon fluconazole exposure

**Figure 5:**
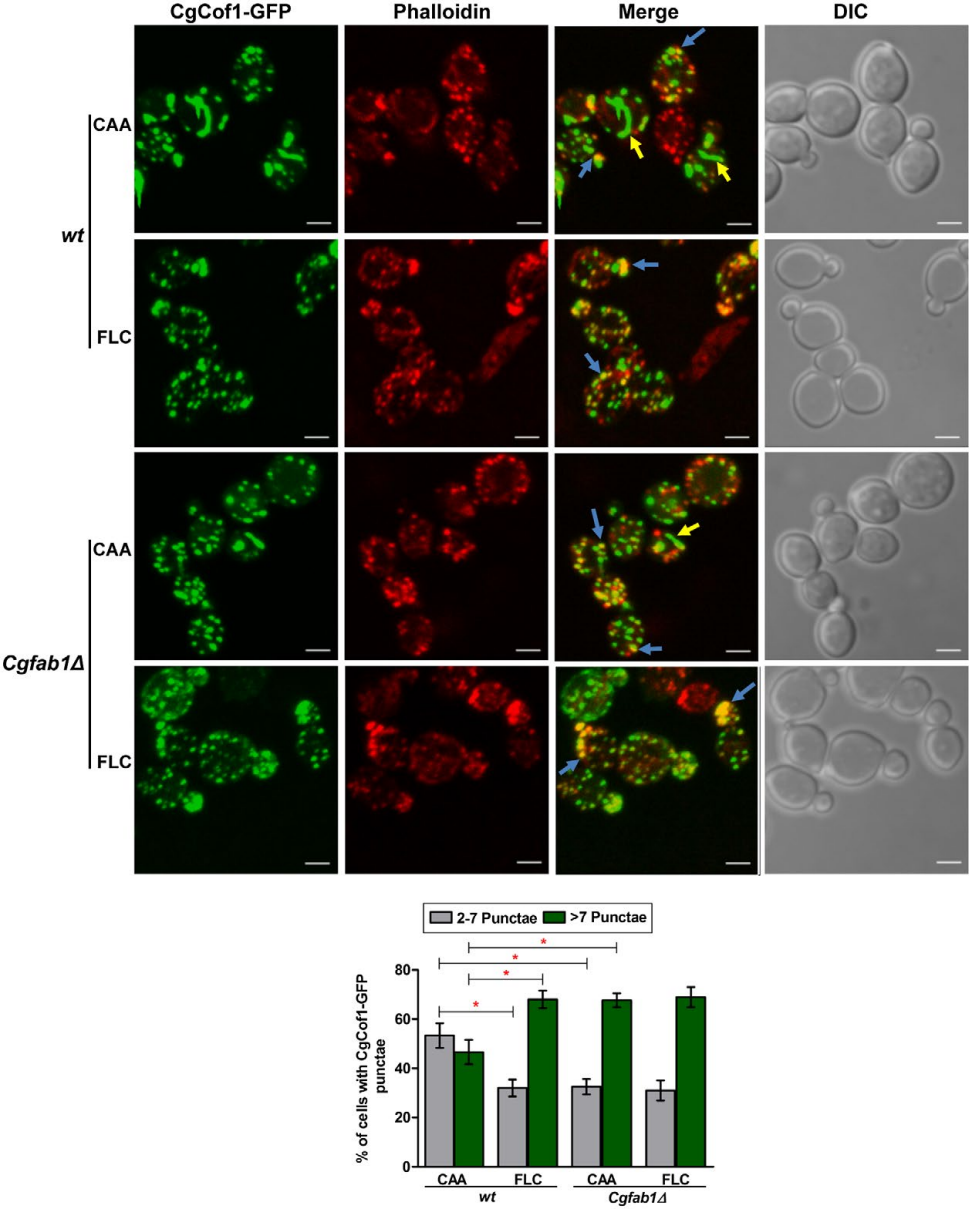
Fluconazole exposure leads to altered localization of the actin‐severing protein, CgCof1. Representative maximum‐intensity projection of Z‐stack fluorescence confocal images showing altered localization of CgCof1‐GFP in 3 h fluconazole (16 µg/ml)‐treated cells. Yellow and blue arrows mark CgCof1‐GFP bar‐like structures and actin colocalized‐CgCof1‐GFP punctae respectively. For each strain, a minimum of 150 cells displaying rhodamine‐phalloidin‐stained actin structures and CgCof1‐GFP punctae were counted, and data (mean ± SEM) are presented, as the percentage of cells containing either two to seven foci or more than seven punctae per cell, underneath the image panels. *p < 0.05; Unpaired, two‐tailed, Student’s t‐test. Bar = 2 µm. [Colour figure can be viewed at http://www.wileyonlinelibrary.com]

To determine how actin structures are depolarized upon fluconazole exposure, we selected two key regulators of the actin structure dynamics, CgCap2 and CgCof1. Cof1 in *S. cerevisiae* is an actin filament‐severing protein, which binds to the pointed end of the actin filament, severs filaments and accelerates actin disassembly (Moon et al., 1993; Mishra et al., 2014). Contrarily, Cap2 binds to the barbed end of the actin filament and halts further actin polymerization in *S. cerevisiae* (Amatruda and Cooper, 1992; Mishra et al., 2014). To investigate the role of CgCap2 and CgCof1 in fluconazole‐induced actin remodeling, we first generated CgCof1‐GFP and CgCap2‐GFP fusion proteins and examined the GFP signal in untreated and fluconazole‐treated cells. CgCof1‐GFP in *wt* was found in the cytosol in two forms: thick bar‐like structures and intense two to seven punctae per cell (Fig. 5). Fluconazole treatment led to an overall increase in the number and a decrease in the size of the CgCof1 punctae, and a diminished number of CgCof1‐bars in *wt* cells (Fig. 5). Interestingly, the *Cgfab1Δ* mutant had a fewer number of CgCof1‐bars and contained more than seven relatively small CgCof1 punctae per cell. Fluconazole treatment had no significant effect on the CgCof1 signal in the *Cgfab1Δ* mutant (Fig. 5). The CgCof1‐GFP punctae partially colocalized with rhodamine phalloidin‐stained actin patches (Fig. 5), indicating association of CgCof1 with actin. The average number (± SEM) of CgCof1‐GFP punctae per cell in *wt*, fluconazole‐treated *wt*,* Cgfab1Δ* and fluconazole‐treated *Cgfab1Δ* cultures was 6.0±0.2, 10.0±0.4, 12.0±0.4 and 11.0±0.4 respectively. The CgCof1‐bars mostly did not costain with rhodamine phalloidin, suggesting that these may represent either G‐actin‐CgCof1 structures or actin‐unassociated/free CgCof1.

**Figure 6:**
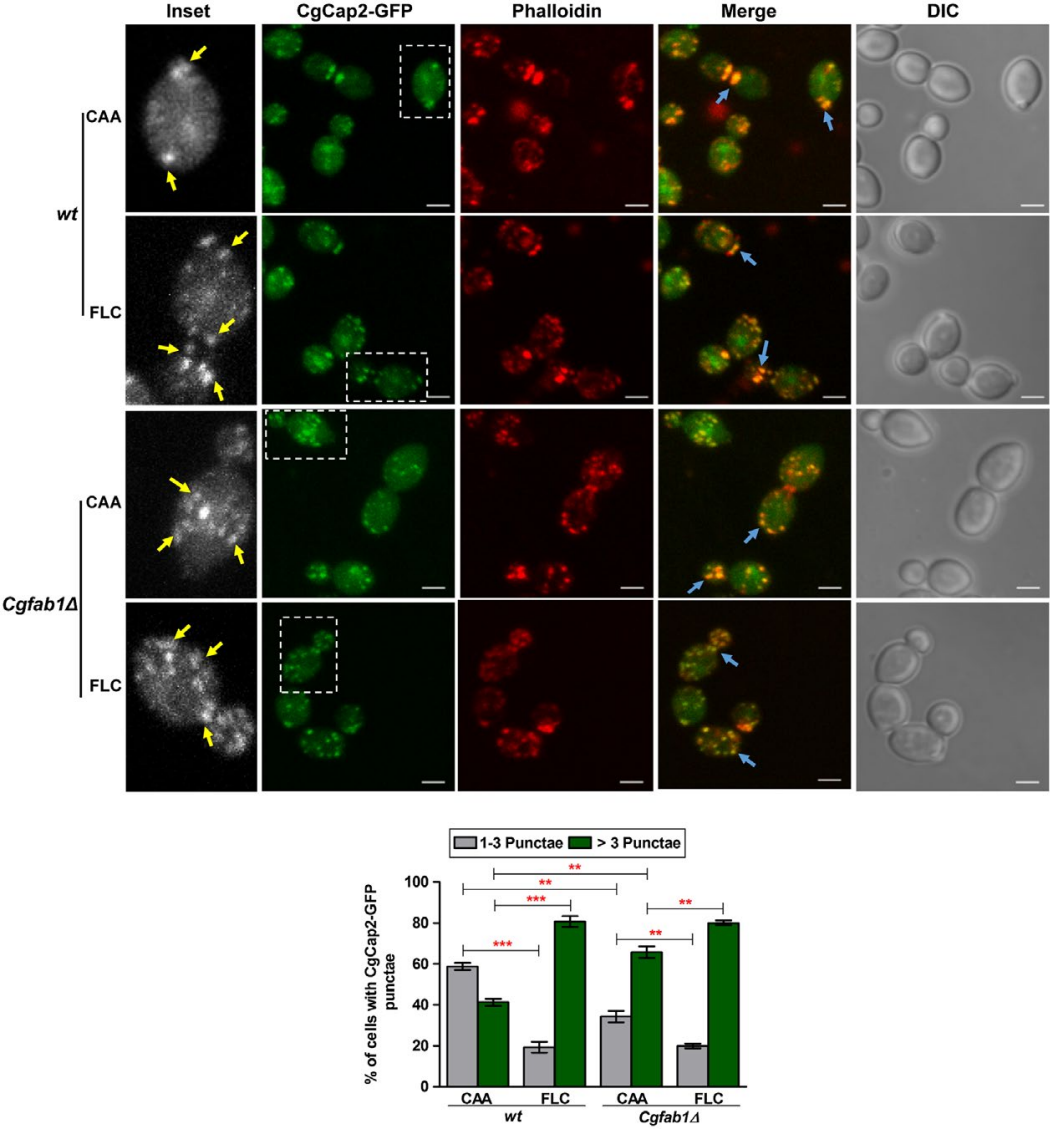
Fluconazole exposure leads to altered localization of the actin‐capping protein, CgCap2. Representative maximum intensity projection of Z‐stack fluorescence confocal images showing altered localization of CgCap2‐GFP in 3 h fluconazole (16 µg/ml)‐treated cells. Yellow and blue arrows mark CgCap2‐GFP punctae and actin colocalized‐CgCap2‐GFP punctae respectively. Inset shows zoomed image of the boxed area. For each strain, a minimum of 150 cells displaying rhodamine‐phalloidin‐stained actin structures and CgCap2‐GFP punctae were counted, and data (mean ± SEM) are presented, as the percentage of cells containing either one to three punctae or more than three punctae per cell, below the image panels. **p < 0.01; ***p < 0.001. Unpaired, two‐tailed, Student's t‐test. Bar = 2 µm. [Colour figure can be viewed at http://www.wileyonlinelibrary.com]

The fluorescent signal of CgCap2‐GFP in *wt* cells was very weak and mostly represented by one to three small punctae that were distributed throughout the cytosol, and also partially co‐localized with rhodamine phalloidin‐stained actin patches (Fig. 6). Both fluconazole treatment and CgFab1 absence resulted in an increase in the number of CgCap2 punctae per cell (Fig. 6). Fluconazole treatment led to a small change in CgCap2 distribution in *Cgfab1Δ* cells (Fig. 6). The average number (± SEM) of CgCap2‐GFP punctae per cell in *wt*, fluconazole‐treated *wt*,* Cgfab1Δ* and fluconazole‐treated *Cgfab1Δ* cultures was 3.0±0.2, 6.0±0.3, 5.0±0.3 and 7.0±0.3 respectively. Altogether, these data indicate that fluconazole exposure changes the distribution pattern of the actin‐modifying proteins.

As actin structures undergo rapid turnover, the smaller but multiple CgCof1 foci, upon fluconazole treatment and *CgFAB1* disruption, may indicate higher CgCof1 activity and faster disassembly/depolymerization of actin structures. Similarly, the higher CgCap2 signal may reflect an increase in the number of depolymerized actin structures. Collectively, these data suggest that fluconazole‐induced depolarization of the actin cytoskeleton may involve enhanced actin depolymerization.

### CgCof1 binds to multiple lipids including PI(3,5)P2

Altered distribution of CgCof1 and CgCap2 in fluconazole‐treated *wt* and *Cgfab1Δ* cells prompted us to examine their functions in actin organization and azole tolerance. To this end, we attempted to generate *CgCOF1* and *CgCAP2* deletion strains. Despite multiple efforts, we could not delete the *CgCOF1* gene from the genome, which may imply its essentiality in *C. glabrata*. Of note, Cof1 is an essential component of the actin cytoskeleton in *S. cerevisiae*, and its null mutants are inviable (Moon et al., 1993). Contrarily, the *Cgcap2Δ* mutant, similar to its *S. cerevisiae* counterpart (Moseley and Goode, 2006), was viable. Morphologically, *Cgcap2Δ* cells were more rounded in shape, and contained mostly actin patches/aggregates with very thin cables, when present, implicating CgCap2 in actin organization (Supporting Information Fig. S5A). The *Cgcap2Δ* mutant also showed azole resistance (Supporting Information Fig. S5B) which suggests that the ability to polymerize actin confers a growth advantage under fluconazole stress.

**Figure 7:**
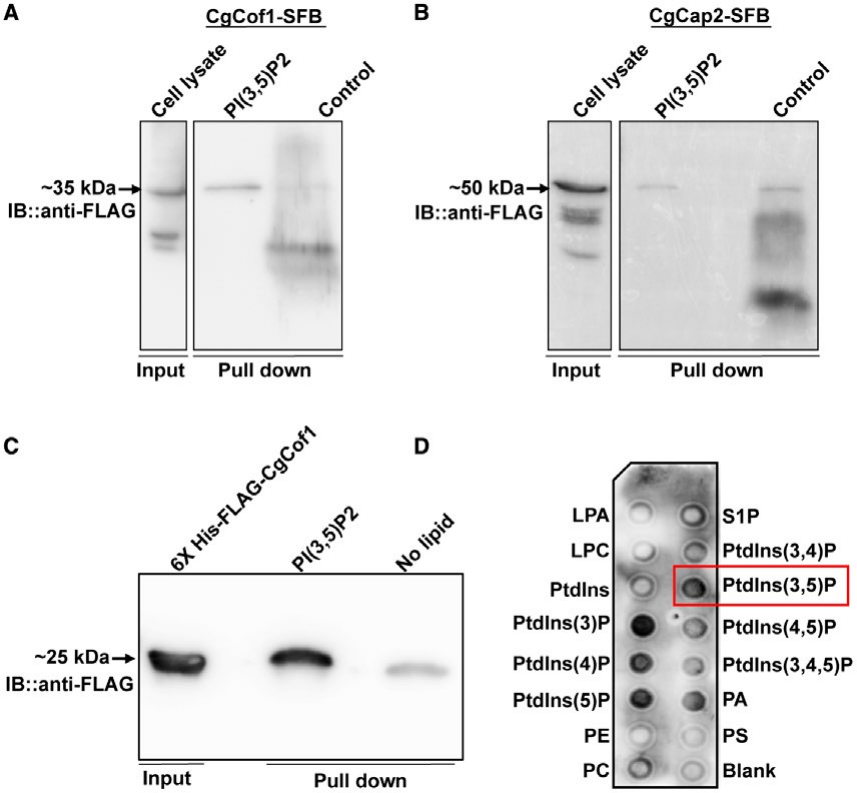
CgCof1 is a PI(3,5)P2‐binding protein. A. Western blot image showing CgCof1‐SFB‐PI(3,5)P2 interaction. B. Western blot image showing lack of specific interaction between CgCap2‐SFB and PI(3,5)P2. CgCap2 signal was of similar intensity in both lipid‐coated and control beads. C. A western blot image showing the interaction of the recombinant 6xHis‐FLAG‐CgCof1 with PI(3,5)P2. Input indicates the 6xHis‐FLAG‐CgCof1 purified from *E. coli*. D. Protein‐lipid overlay assay showing binding of 6xHis‐FLAG‐CgCof1 with phosphatidylinositols and phosphatidic acid lipids. Blank spot contains no lipid. [Colour figure can be viewed at http://www.wileyonlinelibrary.com]

We next asked how CgFab1 could regulate the distribution of CgCof1 and CgCap2. Is it through PI(3,5)P2, the product of the CgFab1 enzyme? Hence, we checked if CgCof1 and CgCap2 interact with PI(3,5)P2. In order to achieve this, CgCof1 and CgCap2 were tagged with SFB (S‐protein‐FLAG epitope‐streptavidin‐binding peptide) at the C‐terminus, and CgCof1‐SFB and CgCap2‐SFB were pulled down from *wt* cell lysates using streptavidin beads. After incubation with PI(3,5)P2‐coated agarose beads, bound proteins were analyzed by western blot (Fig. 7A and B). The PI(3,5)P2 bead‐eluate contained CgCof1‐SFB though a faint CgCof1 signal was also seen in control agarose bead‐eluate (Fig. 7A). Contrarily, the intensity of the CgCap2 signal was similar in both PI(3,5)P2 bead‐ and control bead‐eluates (Fig. 7B). To further verify the binding of CgCof1 to PI(3,5)P2 beads, the 6xHis‐FLAG‐CgCof1 protein was purified from *Escherichia coli*, incubated with either PI(3,5)P2 beads or a strip containing 12 phospholipids and analyzed by western blot. CgCof1 did bind to PI(3,5)P2 on both beads (Fig. 7C) and the membrane (Fig. 7D). Intriguingly, CgCof1 also showed strong binding to four other lipids, namely PI3P, PI4P, PI5P and phosphatidic acid (Fig. 7D). Altogether, these data suggest that CgCof1 binds to different lipid molecules, including PI(3,5)P2, and CgFab1 may modulate actin cytoskeleton organization through regulating the activity of this PI(3,5)P2 interactor. As specific binding of CgCap2 to PI(3,5)P2 was not seen, the role of CgCap2 in actin organization may be independent of PI(3,5)P2 signaling, and warrants further investigation.

### The *Cgfab1Δ* mutant accumulates sterols intracellularly

**Figure 8:**
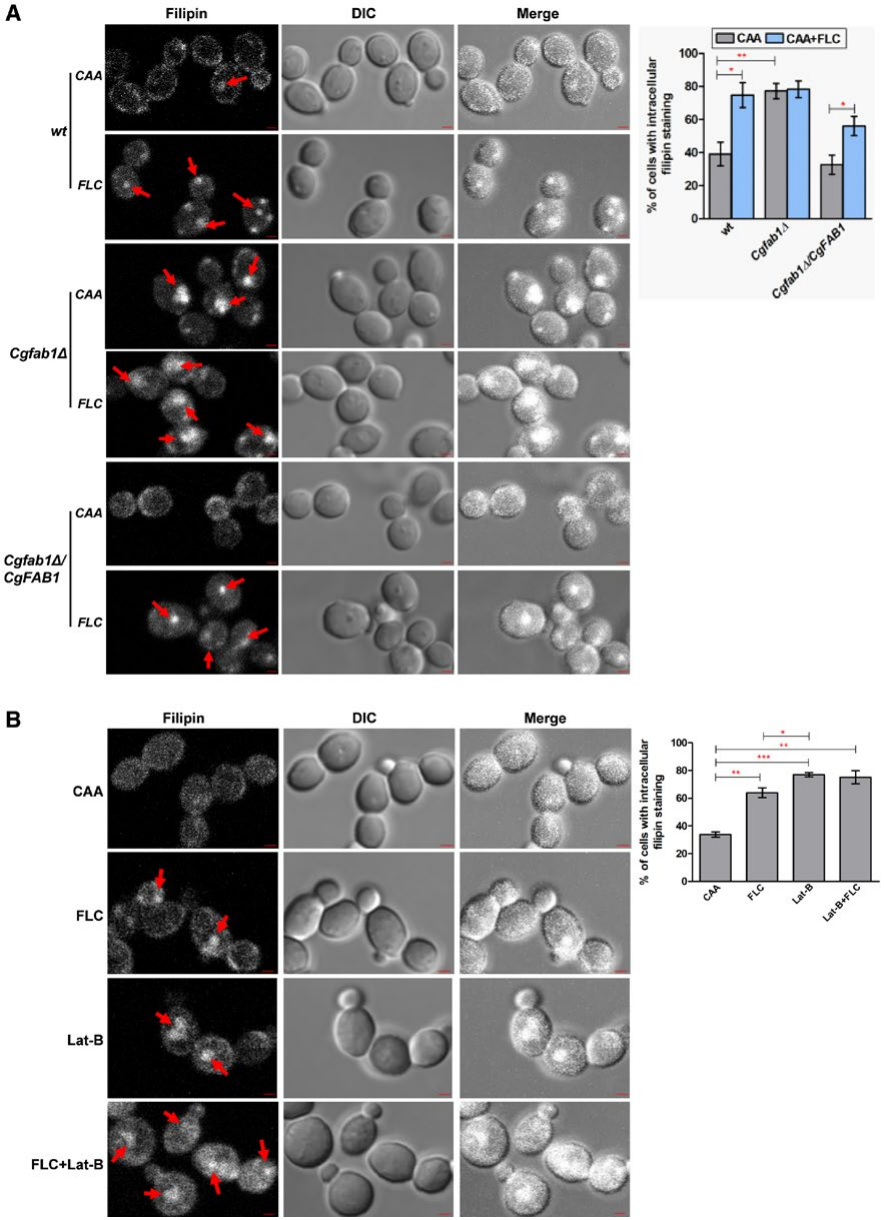
Filipin staining of the *Cgfab1Δ* mutant reveals elevated intracellular sterol accumulation. A. Representative confocal images showing higher intracellular sterol accumulation in the *Cgfab1Δ* mutant and fluconazole (16 µg/ml)‐treated *wt* and *Cgfab1Δ* cells. Red arrows indicate intracellular filipin staining. For each strain, a minimum of 300 cells displaying filipin fluorescence were counted, and data (mean ± SEM) are presented, as the percentage of cells with intracellular filipin signal, on the right side of panels. *p < 0.05; **p < 0.01. Unpaired, two‐tailed, Student's t‐test. Bar = 1 µm. B. Representative confocal images showing intracellular filipin staining in log‐phase *wt* cells treated with fluconazole (16 µg/ml) and Lat‐B (100 µM) for 3 h. For each sample, a minimum of 200 cells displaying filipin fluorescence were counted, and data (mean ± SEM) are presented, as the percentage of cells with intracellular filipin signal, on the right side of panels. *p < 0.05; **p < 0.01, ***p < 0.001. Unpaired, two‐tailed, Student's t‐test. Bar = 1 µm. [Colour figure can be viewed at http://www.wileyonlinelibrary.com]

The last question that we asked was why actin remodeling is required to survive azole stress? It is known that the actin filament network contributes substantially to intracellular transport (Moseley and Goode, 2006). As fluconazole treatment leads to the depletion of ergosterol and accumulation of sterol intermediates (Akins, 2005), we reasoned that trafficking and/or accumulation of sterols may require actin cytoskeletal reorganization. To test this, we performed three experiments. First, we labeled *wt* and *Cgfab1Δ* cells with the fluorescent sterol‐binding dye, filipin. Confocal microscopy revealed that *wt* cells displayed primarily cell surface filipin‐staining pattern, and fluconazole treatment led to a decreased cell membrane signal with a concomitant increase in the intracellular filipin spots (Fig. 8A), which may reflect accumulation of sterol intermediates. Interestingly, sterol staining in the *Cgfab1Δ* mutant was distinct, and 80% of both untreated and fluconazole‐treated *Cgfab1Δ* cells exhibited higher intracellular filipin staining and weaker plasma membrane signal (Fig. 8A) indicating that sterol transport is probably impaired in the mutant.

Second, we examined filipin staining pattern in fluconazole‐ and jasplakinolide‐treated *Cgfab1Δ* cells, and found a modest reduction in the intracellular filipin signal indicative of resumed sterol transport (Supporting Information Fig. S6A). Third, we checked the growth of *wt* and *Cgfab1Δ* cells in medium containing the ergosterol‐binding antifungal amphotericin B (Akins, 2005), and found the *Cgfab1Δ* mutant to exhibit a high level of amphotericin B resistance, which was restored to *wt* levels in the mutant‐complemented strain (Supporting Information Fig. S6B). This result is reflective of altered plasma membrane sterol composition in the *Cgfab1Δ* mutant.

Next, we reasoned that if actin cytoskeletal defect is linked with intracellular sterol accumulation, chemical or genetic abrogation of actin organization should lead to sterol trafficking defects. To address this, we first checked sterol distribution in Lat‐B‐treated *wt* cells and found higher intracellular filipin fluorescent signal (Fig. 8B). However, combined treatment of fluconazole and Lat‐B led to diffuse intracellular filipin signal (Fig. 8B) which may partly be because this combination is fungicidal. We next imaged intracellular sterol accumulation in actin‐defective mutants, *Cgbem2Δ, Cgbnr1Δ*::Tn*7* and *Cgpan1Δ*::Tn*7*, and observed an elevated internal filipin signal (Supporting Information Fig. S6C). Contrarily, the fluconazole‐sensitive *Cgcdr1Δ* mutant (lacks multidrug transporter) mainly exhibited a cell surface filipin staining pattern (Supporting Information Fig. S6C). Altogether, these data support the hypothesis that proper organization of actin networks is pivotal to sterol trafficking in the cell.

## Discussion

Fluconazole toxicity is primarily attributed to the membrane stress due to ergosterol depletion, and accumulation of 14α‐methyl‐3, 6 diol (Akins, 2005). Increased fluconazole efflux is the predominant mechanism of resistance in the inherently less azole‐susceptible pathogenic yeast *C. glabrata* (Tscherner et al., 2011). Here, we demonstrate the multifactorial nature of fluconazole tolerance in *C. glabrata* via identification of 27 new genes involved in cellular response to azoles. Our data underscore conserved features of cellular response to both cell wall‐ and cell membrane‐targeting drugs. Of 26 fluconazole‐sensitive mutants, 12 were caspofungin sensitive and 8 contained higher cell wall chitin. Hence, altered cell wall composition, indicative of activated cell wall compensatory mechanism/s, may contribute to their increased susceptibility to antifungals.

Notably, our mutant collection represents a subset of a library of ~27,000 *C. glabrata* transposon insertion mutants which has recently been screened for altered sensitivity to caspofungin (Rosenwald et al., 2016). Among 26 azole tolerance‐conferring genes identified in our screen, 6 genes, namely *CgSLT2*,* CgMED2*,* CgBEM2*,* CgPDR16, CgSAS2* and *CgITR2*, overlap with a set of 48 caspofungin resistance‐conferring genes identified in that screen (Rosenwald et al., 2016). Importantly, disruption of five of these six genes also led to attenuated growth in our caspofungin susceptibility assays (Fig. 1B). Further, CgBem2 (Rho‐GTPase activator) and CgSlt2 (terminal MAPK) are components of the PKC cell wall integrity pathway (Borah et al., 2011), and mutants lacking key PKC cascade constituents are known to exhibit caspofungin sensitivity (Schwarzmuller et al., 2014). Together, these findings highlight the pivotality of PKC signaling in response to both azole and echinocandin antifungals in *C. glabrata*. It is worth mentioning that the PKC‐mediated cell wall integrity pathway may also participate in sensing and relaying the signal for actin remodeling in response to fluconazole, as Slt2 has been implicated in actin repolarization after heat stress in *S. cerevisiae* (Delley and Hall, 1999).

Phosphoinositides regulate several key cellular processes, including vesicle‐mediated membrane trafficking, actin cytoskeleton organization and cytokinesis (Odorizzi et al., 2000; McCartney et al., 2014). Among seven known phosphoinositides, PI(3,5)P2 represents only about 0.1% of total PI, and is required for vacuolar morphology and function, multivesicular body sorting and retrograde traffic from the vacuole to the Golgi in *S. cerevisiae* (McCartney et al., 2014). Fab1, a PI3P‐binding FYVE domain‐containing kinase, converts PI3P to PI(3,5)P2, and PI(3,5)P2 cellular levels are maintained by Fab1‐mediated synthesis and Fig4 phosphatase‐mediated degradation (McCartney et al., 2014). Here, we demonstrate for the first time that CgFab1 regulates antifungal tolerance and vacuole homeostasis in *C. glabrata*. Fluconazole exposure also led to a 2.5‐fold decrease in *CgFAB1* transcript levels. Altogether, our data establish CgFab1 as a key determinant of basal azole tolerance owing to its role in actin cytoskeletal organization.

Further, we show that fluconazole triggers actin restructuring with depolarization of patches and loss of cables, and these imbalances in actin network contribute to azole susceptibility in *C. glabrata*. Notably, heat shock and osmotic stress are known to result in the disappearance of actin cables in *S. cerevisiae* (Chowdhury et al., 1992; Lillie and Brown, 1994). Dynamic actin cytoskeleton reconfiguration has been associated with cell cycle progression, cell motility and endocytosis (Moseley and Goode, 2006). Since cell cycle profiles of untreated and fluconazole‐treated wild‐type cells have previously been reported to be similar (Borah et al., 2011), actin structure remodeling is unlikely to be due to cell cycle arrest in our study.

Lastly, our data suggest that actin network restructuring, at least in part, modulates sterol transport. Diminished sterol trafficking, probably owing to inadequate actin connections, and impeded ergosterol biosynthesis may contribute to intracellular sterol accretion. This notion is strengthened by elevated sterol accumulation in actin‐defective mutants, including the *Cgfab1Δ* mutant. Importantly, transcript levels of ergosterol biosynthetic enzyme‐encoding genes and total sterol content were similar between *Cgfab1Δ* and *wt* cells (Supporting Information Fig. S6D and data not shown); hence, impaired sterol trafficking is likely to account for intense internal filipin signal in the mutant. However, the off‐target effects of PI(3,5)P2 loss need to be precluded.

How does CgFab1 kinase modulate actin structure? The answer possibly lies in PI(3,5)P2‐dependent regulation of actin‐modifying functions of the actin‐binding proteins. The actin depolymerizing protein CgCof1 appears to be one such protein. Through homology modeling analysis, we predicted PI(3,5)P2‐binding residues, viz, Lys36, Arg96, Ser103 and Ser104 in CgCof1 (Supporting Information Fig. S7). These amino acids were also predicted to interact with actin, and their substitution with alanine abolished the actin binding of CgCof1. Nonetheless, the functional relevance of the predicted binding amino acids in azole tolerance in vivo is yet to be examined.

Further, a critical role for PI(3,5)P2 in azole tolerance was strengthened by azole sensitivity phenotype of the *Cgvac7Δ* and *Cgvac14Δ* mutants (Supporting information Fig. S8A), which, similar to their *S. cerevisiae* counterparts, may contain lower PI(3,5)P2 levels. Of note, PI(3,5)P2 levels in *S. cerevisiae* are maintained by Fab1‐mediated synthesis and Fig4 [PI(3,5)P2‐specific 5‐phosphatase]‐mediated degradation (Gary et al., 2002; Botelho et al., 2008). The *S. cerevisiae* Fab1 enzyme is known to be activated by Vac7 (vacuolar segregation protein) and Vac14 (scaffolding adaptor protein), and Vac14 forms a PAS complex with Vac7, Fab1 and Fig4 (Gary et al., 2002; Botelho et al., 2008). Consistently, *vac7Δ* and *vac14Δ* mutants in *S. cerevisiae* have undetectable levels of PI(3,5)P2 (Gary et al., 2002; Rudge et al., 2004). *C. glabrata Cgvac7Δ* and *Cgvac14Δ* mutants displayed large vacuoles (Supporting information Fig. S8B), indicating a vacuole homeostasis defect. These mutants also likely have lower cellular PI(3,5)P2 levels. Hence, elevated susceptibility of *Cgvac7Δ* and *Cgvac14Δ* mutants to azole antifungals (Supporting information Fig. S8A) and its rescue by jasplakinolide (Supporting information Fig. S8C) underscore the role of the PI(3,5)P2 lipid in azole tolerance, and render the possibility of CgFab1 specifically interacting with azoles to alter actin dynamics unlikely.

Actin cable turnover in *S. cerevisiae* requires cofilin function, and based on the availability of other actin‐binding proteins, Cof1 can both impede and enhance actin polymerization (Moseley and Goode, 2006; Mishra et al., 2014). Based on our data, we propose that fluconazole exposure diminishes CgFab1 activity which results in a reduction in intracellular PI(3,5)P2 levels. Owing to the competitive binding of actin and PI(3,5)P2 to CgCof1, this is likely to lead to increased CgCof1 activity and actin depolymerization, thereby enhancing actin dynamics. Of note, ADF/cofilin proteins bind to multiple PI(4,5)P2 molecules in a cooperative manner (Zhao et al., 2010), and PI(4,5)P2 negatively regulates actin binding of Cof1 (Zhao et al., 2010). Additionally, Cof1 in *S. cerevisiae* is implicated in the mitochondrial function‐dependent MDR (Kotiadis et al., 2012).

Fluconazole exposure and *CgFAB1* disruption altered the distribution of both CgCof1 and CgCap2 inside the cell. In *S. cerevisiae*, Cap2 forms a heterodimer with another subunit Cap1, and localizes on actin patches (Amatruda and Cooper, 1992; Moseley and Goode, 2006). Cap2 also binds to the barbed ends of actin filaments to impede addition as well as dissociation of actin subunits (Amatruda and Cooper, 1992; Moseley and Goode, 2006). The loss of actin cables and decreased fluconazole susceptibility in the *Cgcap2Δ* mutant implicate CgCap2 in actin filament stabilization and azole tolerance respectively. However, despite altered CgCap2 localization in the *Cgfab1Δ* mutant, we presently do not know the relationship, if any, between CgFab1 and CgCap2 activity, and this aspect needs to be investigated further.

In conclusion, we report a hitherto unknown physiological effect of fluconazole on actin reorganization in *C. glabrata* which may open the way toward exploring new combinatorial therapeutic antifungal strategies.

## Experimental procedures

### Strains and media

*C. glabrata* wild‐type and mutant strains, which are derivatives of the BG2 strain, were routinely cultured in YPD medium at 30°C. *C. glabrata* transformants were grown either in CAA medium or in the synthetically defined YNB medium. Bacterial strains lacking or carrying plasmids were grown in LB medium and LB medium containing ampicillin/kanamycin, respectively, at 37°C. Logarithmic (log)‐phase *C. glabrata* cells were collected after 4 h growth of overnight cultures in the fresh medium.

### *C. glabrata* gene disruption and cloning

*C. glabrata fab1∆*,* cap2∆*,* vac7∆* and *vac14∆* strains were created using the homologous recombination‐based strategy as described previously (Borah et al., 2011). For generation of the reconstituted strain, *CgFAB1* (*CAGL0K10384g*, 6.31 kb) was PCR‐amplified from the wild‐type genomic DNA, cloned downstream of the *PGK1* promoter in BamHI‐SalI restriction sites in the pGRB2.2 (pRK74) plasmid and was expressed in the *Cgfab1∆* mutant. For the creation of CgFab1 kinase‐dead version, the region (2.51 kb) encoding the predicted lipid kinase domain was amplified with mutagenic primers in two halves sharing 45 bp complementary sequence followed by fusion PCR. The fusion PCR product contained two glycine residues at 1867 and 1870 *nt* position in the *CgFAB1* ORF (open reading frame) mutated to valine residues, and the mutated region was used to replace the corresponding region in the parental plasmid (pRK1033). Mutations were confirmed by sequencing. For generation of C‐terminal‐GFP fusion proteins, PCR‐amplified *CgFAB1*,* CgCAP2* (*CAGL0I05214g*, 0.81 kb) and *CgCOF1* (*CAGL0E04048g*, 0.91 kb) ORFs were cloned downstream and upstream of the *PGK1* promoter and GFP‐encoding region, respectively, in the pGRB2.3 plasmid. *CgFAB1*,* CgCAP2* and *CgCOF1* were cloned in BamHI‐XmaI, SpeI‐XmaI and SpeI‐BamHI, respectively, in the pGRB2.3 plasmid. For PI(3,5)P2 ‐binding assays, *CgCOF1* and *CgCAP2* were cloned downstream of the *PDC1* promoter and upstream of the SFB (S protein, FLAG and streptavidin‐binding peptide) tag between SpeI‐BamHI and SpeI‐XmaI sites, respectively, in the pRK1349 plasmid. For acquired azole resistance studies, the *CgPDR1‐GOF* allele was integrated into the genome of the *Cgfab1∆* mutant and *wt* strain, as described previously (Borah et al., 2014). Strains and plasmids used in this study are listed in Table S2 (Supporting Information). The sequence of primers used in this study is listed in Table S3 (Supporting Information).

### Stress susceptibility assays

The *C. glabrata* Tn*7* insertion mutant library screen and identification of Tn*7* insertion position in the mutant genome were carried out as described previously (Borah et al., 2011). *C. glabrata* strains were considered as fluconazole‐sensitive if they displayed attenuated growth in fluconazole at a concentration of 16 µg/ml. In our assays, the growth of the wild‐type strain was found to be inhibited in the presence of 32 µg/ml concentration of fluconazole. Hence, *C. glabrata* strains growing better than the wild‐type strain in the medium containing 32 µg/ml fluconazole were classified as fluconazole‐resistant. The susceptibility of strains to fluconazole, metal ions, cell wall and cell membrane stress and oxidative stress was assessed in the solid medium through spot assay. For serial dilution spotting analysis, *C*. *glabrata* strains were grown overnight in YPD medium, OD_600_ was normalized to 1.0 and cultures were diluted serially 10‐fold in PBS. Three microliters of each dilution was spotted on the appropriate medium and growth was recorded after 1–2 days of incubation at 30ºC. The susceptibility of Tn*7* insertion mutants was checked toward fluconazole (FLC, 16, 32 and 64 µg/ml), cycloheximide (CHX, 1 µg/ml), caffeine (10 mM), sodium dodecyl sulfate (SDS, 0.05%), geldanamycin (GLD, 25 µM), ethylenediaminetetraacetic acid (EDTA, 200 µg/ml), ethylene‐bis(oxyethylenenitrilo)tetraacetic acid (EGTA, 20 mM) and glycerol as a sole carbon source (YPG, 3%). Sorbitol was used at a concentration of 1 M to rescue growth defects. Liquid growth analyses with caspofungin, EGTA, amphotericin B, Lat‐B and jasplakinolide were performed in a 96‐well plate wherein each well was inoculated with an overnight culture equivalent to 0.2 OD_600_ to a final volume of 100 μl. After 24 h incubation at 30°C, cultures were diluted 100‐, 300‐ and 500‐fold, and 3 μl of each dilution was spotted on YPD medium. Plates were photographed after 24–48 h incubation at 30°C.

### Chitin estimation

Log‐phase *C. glabrata* cultures were collected, PBS‐washed and normalized to 2.0 OD_600._ After 15 min incubation with calcofluor white (2.5 µl, 10 mg/ml stock) at room temperature in the dark, cells were washed twice with PBS and ~ 50,000 cells were analyzed via flow cytometry (BD FACS ARIA III). Mean fluorescence intensity ratio reflects mutant/wild‐type fluorescence intensity values.

### Quantitative real‐time PCR (qPCR)

Log‐phase *wt* and *Cgfab1Δ* cultures were inoculated at an OD_600_ of 0.1 in the YPD medium lacking or containing fluconazole (16 µg/ml) and grown for 4 h at 30°C. Total RNA was extracted using the acid phenol extraction method and digested with deoxyribonuclease I to eliminate any DNA contamination. DNase I‐digested RNA was used to synthesize cDNA using the SuperScript III First‐Strand Synthesis System for RT‐PCR. qPCR was performed with the MESA GREEN qPCR mastermix using primers specific for *CgCDR1*,* CgPDR1*,* CgFAB1* and the housekeeping gene *CgTDH3* [codes for glyceraldehyde‐3‐phosphate dehydrogenase (GAPDH)], whose expression was not changed upon fluconazole treatment. C_T_ (cycle threshold) values of *CgFAB1*,* CgCDR1* and *CgPDR1* genes were normalized against the corresponding C_T_ value obtained for the *CgTDH3* gene under a similar condition, and the fold‐change in expression in fluconazole‐treated samples compared to untreated cultures was determined by the comparative C_T_ (2^–∆∆C^_T_) method. Transcript levels of *CgERG1*,* CgERG3*,* CgERG4*,* CgERG6* and *CgERG11* genes were measured in log‐phase cultures of *wt* and *Cgfab1Δ* mutants using the C_T_ method.

### Fluorescent staining

Confocal imaging was done at room temperature using the point scanning confocal system (Zeiss LSM 700) equipped with 100X/1.44 NA objective. Localization studies of CgFab1‐GFP, CgCof1‐GFP and CgCap2‐GFP were performed on live cells. For the study of actin structures and cellular distribution of CgCof1‐GFP and CgCap2‐GFP, Z‐stack images were acquired throughout the cell at 0.25 µM intervals. Fluorescent images were deconvolved, and a subset (6–10 images) of the entire Z‐stack was projected into a single image of maximum intensity using the Zen Blue software. A minimum of 150 small‐budded cells, wherein the ratio of bud volume to total cell volume was less than 30%, were counted for each strain/condition to determine the percentage of cells that either contained both actin cables and patches or lacked visible actin cables. The number of CgCof1‐GFP punctae and CgCap2‐GFP punctae was determined in a minimum of 150 cells for each strain/condition. Further, due to the very close proximity of CgCof1‐GFP punctae in small buds, the foci were counted only in mother cells for accuracy. For vacuole staining, YPD‐grown log‐phase cells were collected, washed and suspended in YPD medium containing FM4‐64 (16 µM). After 30 min incubation in the dark at 30°C, cells were washed and grown in YPD medium for 90 min. Cells were collected, washed, suspended in YNB/CAA medium and imaged.

For rhodamine‐conjugated phalloidin staining, log‐phase *C. glabrata* cells were grown for 3 h either in YPD medium or YPD medium containing fluconazole (16 µg/ml). Cells were washed twice with phosphate buffer (0.1 M, pH 7.0) and incubated with formaldehyde (3.7%) for 60 min at room temperature. Cells were washed and incubated in the phosphate buffer containing Triton‐X (0.1%) and rhodamine‐conjugated phalloidin (165 nM). After 60 min incubation in the dark at 4˚C with rocking, cells were collected, washed and placed on the slide in the mounting medium. For colocalization studies, log‐phase cultures were treated with 16 µg/ml fluconazole for 3 h, cells were collected and suspended in SHA buffer [100 mM HEPES (pH 7.4), 1 M sorbitol, 5 mM sodium azide, 0.2% Triton X‐100] containing rhodamine phalloidin (165 nM). After 10 min incubation at 30˚C in the dark, the cell suspension was incubated on ice for 15 min, washed with PBS and imaged. For filipin staining, log‐phase cells were incubated with freshly prepared filipin (100 μg/ml) for 5 min, collected and imaged immediately using the Vectashield mounting medium. Image acquisition and analysis were performed using ZEN software.

### Vacuolar‐ATPase activity determination

YPD‐grown log‐phase *C. glabrata* cells were collected and vacuolar membranes were isolated using a 0%–4% Ficoll gradient as described previously (Bairwa et al., 2014). H^+^‐ATPase activity in purified vacuolar membranes was measured in the buffer [MES/Tris/HCl (100 mM; pH 6.9), MgCl_2_ (20 mM) and KCl (100 mM)] containing 10 μg protein and 5 mM ATP. NaN_3_ (2 mM), Na_3_VO_4_ (200 μM) and (NH_4_)_2_MoO_4_ (0.2 mM) were used to inhibit residual activities from mitochondrial ATPases, plasma membrane H^+^‐ATPase and phosphatases respectively. After incubation at 30^°^C for 30 min, a solution containing SDS, H_2_SO_4_, (NH_4_)_2_MoO_4_ and ascorbic acid was added to stop the reaction and liberated Pi was measured at *A*_750_. ATPase activity was expressed in micromoles of Pi (calculated from the KH_2_PO_4_ standard curve) released per milligram of protein per minute. ATPase activity measured in the presence of sodium azide (2 mM), sodium orthovanadate (200 µM) and bafilomycin A (20 µM; a specific inhibitor of the vacuolar H^+^‐ATPase) was subtracted from the activity measured without bafilomycin to calculate the V‐ATPase activity. Vacuolar membrane ATPase activity was plotted as % ATPase activity difference in the absence of bafilomycin A minus the activity in the presence of bafilomycin A.

### Purification of recombinant CgCof1

The *CgCOF1* coding sequence, amplified using oligos OgRK2739 and OgRK2040 from the *wt C. glabrata* gDNA, was cloned in EcoRI and XhoI sites in the pET‐28a bacterial expression plasmid. This construct carried hexa‐histidine‐ and FLAG‐tag sequence at the N‐terminus. The recombinant CgCof1 was purified from the *E. coli* BL21‐Codon Plus (DE3) strain with Ni‐NTA chromatography. Western blot analysis with anti‐FLAG antibody (1:10000 dilution; Sigma; #F1804) revealed that the purified CgCof1 protein migrated as an ~25 kDa band in SDS‐PAGE, which is larger than its predicted size of 17 kDa.

### Yeast protein extraction

*Candida glabrata wt* cells expressing CgCof1‐SFB (26 kDa) or CgCap2‐SFB (41 kDa) were grown to log phase in CAA medium at 30^°^C for 5 h and collected. Cells were lysed in lysis buffer (20 mM Tris‐HCl pH 8.0, 100 mM NaCl, 1 mM EDTA, 0.5% NP‐40 containing 1 mM sodium orthovanadate, 1 mM PMSF, 10 mM sodium fluoride and 1x protease inhibitor) using glass beads (0.45 mm) for five intervals of 1 min each, with each interval followed by a 5 min incubation on ice. The cell lysate was centrifuged at 15,000 rpm for 15 min, and the soluble protein fraction was collected. The extracted proteins (10 mg) were incubated with streptavidin‐sepharose beads (150 µl; GE Healthcare; #17‐5113‐01) overnight at 4^o^C using a rotor mixer. Beads were washed four times with lysis buffer and incubated in a solution of biotin (2 mg/ml) for 2 h at 4^o^C to elute proteins off the beads. Cof1‐SFB (30 µg) and Cap2‐SFB (30 µg) from this pull‐down assay were used for the PI(3,5)P2‐binding assay. Notably, both proteins displayed molecular weight larger than their predicted size, with CgCof1‐SFB and CgCap2‐SFB bands corresponding to ~ 35 and ~ 50 kDa, respectively, in SDS‐PAGE.

### PI(3,5)P2‐binding assay

For lipid‐binding assays, PI(3,5)P2‐coated (Echelon Biosciences; #P‐B035a) and control (No lipid Echelon Biosciences; #P‐B000) beads were used. Briefly, lipid and control beads (50 µl each) were incubated in the blocking buffer (PBST; PBS with 0.1% Tween‐20) containing BSA (3%) overnight at 4^°^C and collected. These beads were further incubated with either proteins (30 µg) pulled down from *C. glabrata* cells or the recombinant CgCof1 (25 µg) in PBST buffer containing sodium orthovanadate (1 mM), PMSF (1 mM), sodium fluoride (10 mM) and protease inhibitor cocktail (1×) overnight at 4^°^C, washed four times with PBST and suspended in 2X Laemmli buffer (120 mM Tris‐HCl, 20% glycerol, 4% SDS, 5% β‐mercaptoethanol, 0.2% bromophenol blue). Bead‐bound proteins were eluted by boiling at 95^°^C for 5 min, resolved on SDS‐PAGE and immunoblotted with the anti‐FLAG antibody (1:5000 dilution). For the PIP strip lipid‐protein binding assay, the hydrophobic membrane, containing 100 pmol of eight phosphoinositides and seven other lipid molecules (Echelon Biosciences; #P‐6001), was blocked with PBST containing nonfat dry milk (1%) overnight at 4^°^C. The FLAG‐tagged CgCof1 (25 µg) protein was incubated with the blocked membrane for 2 h at 4^°^C, washed four times with PBST and probed with the anti‐FLAG antibody (1:5000 dilution). PtdIns, LPA, LPC, PE, PC, S1P, PA and PS refer to phosphatidylinositol, lysophosphatidic acid, lysophosphatidylcholine, phosphatidylethanolamine, phosphatidylcholine, sphingosine‐1‐phosphate, phosphatidic acid and phosphatidylserine, respectively, on the membrane.

### Other procedures

Rhodamine 6G efflux, western blot and colony blot analysis were performed as described previously (Borah et al., 2011; Rai et al., 2015).

### Statistical analysis

Statistical analysis was performed using the GraphPad Prism software. The two‐tailed Student’s t‐test was used for intergroup comparisons.

## Conflict of interest

The authors declare that they have no conflict of interest.

## Author contributions

PB, RS, DKC and RK conceived and designed the study. PB, RS, DKC and SB performed experiments and acquired data. PB, DKC, RS, SB and RK analyzed data. PB, DKC and RK prepared figures and wrote the manuscript.
