# Supplementary material for: Fluconazole‐induced actin cytoskeleton remodeling requires phosphatidylinositol 3‐phosphate 5‐kinase in the pathogenic yeast Candida glabrata
Source: Mol Microbiol. 2018 Oct 3;110(3):425–43. doi: 10.1111/mmi.14110 (PMC6221164; doi:10.1111/mmi.14110)
Supplement: Supplementary file 1 [file MMI-110-425-s001.pdf]

Supporting Information

Fluconazole-induced actin cytoskeleton remodeling requires phosphatidylinositol 3-phosphate 5-kinase in the pathogenic yeast *Candida glabrata*

Priyanka Bhakt, Raju Shivarathri, Deepak Kumar Choudhary, Sapan Borah and Rupinder Kaur

Supplementary material includes three tables (Supplementary Tables 1-3) and eight figures (Supplementary Figures 1-8).

Supplementary Tables

Table S1: Tn*7* mutants identified in the genetic screen for altered fluconazole susceptibility profiles.

Table S2: List of strains and plasmids used in the study

Table S3: List of primers used in the study

Legends to Supplementary Figures

Figure S1: The *Cgfab1Δ* mutant responds to azole stress through activation of the MDR pathway.

- **A.** qPCR-based measurement of *CgCDR1* and *CgPDR1* transcript levels in log-phase *wt* and *Cgfab1Δ* cultures. Data (mean ± SEM, n = 3-5) represent fold change in expression in fluconazole-treated samples compared to untreated cultures. **, p<0.01; ***, p<0.001, paired two-tailed Student's t-test.
- **B.** Glucose-induced rhodamine 6G (R6G) efflux by 4 h fluconazole-treated and untreated log phase cultures. Glucose-starved cells were preloaded with 10 µM R6G for 2 h at 30°C and

the supernatant fluorescence, read-out of R6G extracellular concentration, was measured after 20 min of glucose (2 mM) addition. Data (mean ± SEM, n = 6-8) are expressed in arbitrary units (AU).

C. Serial dilution cell spotting assay showing azole resistance-conferring ability of the *CgPDR1-GOF* (*CgPDR1^{L280F}*) allele in the *Cgfab1Δ* mutant. Fluconazole was used at a concentration of 16 µg/ml (FLC-16) and 64 µg/ml (FLC-64).

Figure S2: CgFab1 is required for vacuole functions.

A. Schematic illustration of domain structure of the CgFab1 protein as predicted by the NCBI Conserved Domain tool. The diagram is not drawn to scale.

B. Serial dilution cell spotting assay illustrating sensitivity of the *Cgfab1Δ* mutant to manganese chloride ($MnCl_2$; 3 mM) and zinc chloride ($ZnCl_2$; 8 mM).

C. Western blot analysis of whole-cell extracts (40 µg) from indicated 12 h YPD-grown *C. glabrata* cells.

D. Serial dilution spotting analysis illustrating CgFab1-GFP-mediated complementation of fluconazole (16 µg/ml; FLC), clotrimazole (2 µg/ml; CTZ) and manganese chloride (3 mM; $MnCl_2$) sensitivity of the *Cgfab1Δ* mutant.

E. Serial dilution spotting analysis illustrating growth of *Cgfab1Δ*, *Cgvps15Δ*, *Cgvps34Δ* and *Cgyps1-11Δ* mutants in the CAA medium lacking or containing fluconazole (16 µg/ml; FLC) and clotrimazole (2 µg/ml; CTZ). Plate images were captured after growth at 30ºC for 1-2 days.

Figure S3: Fluconazole treatment downregulates *CgFAB1* gene expression.

A. qPCR-based measurement of *CgFAB1* transcript levels in fluconazole-treated *wt* cultures. Data (mean ± SEM, n = 5) were normalized against the *CgGAPDH* mRNA control, and

represent fold change in expression in fluconazole-treated samples (FLC) compared to untreated cultures (CAA). **, p<0.01, paired two-tailed Student's t-test.

B. Jasplakinolide treatment did not rescue large-vacuole phenotype of the *Cgfab1Δ* mutant. Log phase *Cgfab1Δ* cultures were either grown in the CAA medium (CAA) or CAA medium containing 750 ng/ml of jasplakinolide (JSP) at 30°C. After 2 h, cells were collected, stained with FM4-64 and imaged using confocal microscopy. Bar = 2 μm.

Figure S4: Varied azole susceptibility of the clinical isolates of *C. glabrata*. Serial dilution cell spotting analysis showing fluconazole susceptibility of *C. glabrata* clinical isolates. 4 isolates showed *wt*-like sensitivity to fluconazole (16 µg/ml; FLC-16), while 6 isolates displayed resistance to fluconazole [(64 µg/ml; FLC-64 (FLC-R)].

Figure S5: CgCap2 is required for organization of the actin cytoskeletal network.

A. Representative confocal images showing loss of actin cables and rounded morphology in rhodamine-phalloidin-labelled *Cgcap2Δ* cells. White and yellow arrows mark actin cables and patches, respectively. Bar = 1 µm.

B. Serial dilution cell spotting assay showing azole resistance in the *Cgcap2Δ* mutant. Fluconazole [(FLC-16 (16 µg/ml), FLC-32 (32 µg/ml) and FLC-64 (64 µg/ml)], and clotrimazole [(CTZ-2 (2 µg/ml) and CTZ-6 (6 µg/ml)] were used. Ectopic expression of CgCap2-GFP reverted the azole resistance in the *Cgcap2Δ* mutant to *wt*-levels.

Figure S6: Actin cytoskeletal-defective mutants display increased intracellular sterol accumulation.

A. Representative confocal images showing decreased intracellular sterol accumulation in *Cgfab1Δ* cells treated with jasplakinolide (750 ng/ml; JSP) for 2 h. Fluconazole concentration used was 16 µg/ml. For each sample, a minimum of 200 cells displaying

filipin fluorescence were counted, and data (mean ± SEM) are presented, as the percentage of cells with intracellular filipin signal, on the right side of panels. *, $p<0.05$; **, $p<0.01$. Unpaired, two-tailed, Student's t-test. Bar = 1 µm.

B. CAA liquid growth assay-based analysis of amphotericin B (3 µg/ml) resistance in the *Cgfab1Δ* mutant.

C. Representative confocal images showing higher levels of intracellular sterols in filipin-stained *Cgbem2Δ*, *Cgbnr1Δ::*Tn*7* and *Cgpan1Δ::*Tn*7*, mutants. The *Cgcdr1Δ* mutant did not accumulate sterols intracellularly. Red arrows indicate intracellular filipin staining. Bar = 1 µm.

D. qPCR-based measurement of indicated *CgERG* gene transcript levels in *wt* and *Cgfab1Δ* cultures. Data (mean ± SEM, n = 3-5) were normalized against the *CgGAPDH* mRNA control, and represent fold change in expression in the *Cgfab1Δ* mutant compared to the *wt* strain.

Figure S7: Molecular docking analysis of CgCof1 with PI(3,5)P2 and CgAct1 using the Autodock Vina and Z-DOCK servers, respectively. CgAct1 and PI(3,5)P2 showed binding to same residues (Lys36, Arg96, Ser103 and Ser104) in CgCof1.

Figure S8: *Cgvac7Δ* and *Cgvac14Δ* mutants contain large vacuole and are azole sensitive.

A. Serial dilution cell spotting assay showing sensitivity of *Cgvac7Δ* and *Cgvac14Δ* mutants towards azoles. Fluconazole (FLC), clotrimazole (CTZ) and ketoconazole (KTZ) were used at a concentration of 16 µg/ml, 2 µg/ml and 4 µg/ml, respectively.

B. Representative confocal images of FM4-64 stained, YPD-grown log-phase cultures of indicated *C. glabrata* strains. Bar = 2.0 µm.

C. Liquid growth assay-based analysis of jasplakinolide (750 ng/ml; JSP)-mediated rescue of fluconazole (48 µg/ml; FLC) sensitivity.

Table S1: Tn*7* mutants identified in the genetic screen for altered fluconazole susceptibility profiles.

Gene name	CAGL ID	Tn*7* insertion position (*nt*)	ORF length (*nt*)	Function of the *S. cerevisiae* ortholog
**Mutants identified in the fluconazole resistance screen**				
**Cellular protein modification process**				
SET2	*CAGL0C00297g*	1428	2151	Histone methyltransferase with a role in transcriptional elongation; methylates H3 lysine 36 (H3K36)
HST1	*CAGL0C05357g*#	802	1530	NAD^+-dependent histone deacetylase
SET4	*CAGL0G04499g*	36	1053	Protein of unknown function; contains a SET domain
**Organelle organization**				
HMI1	*CAGL0I07623g*	902	2136	Mitochondrial inner membrane localized ATP-dependent DNA helicase
CCE1	*CAGL0L05346g*	118	996	Mitochondrial cruciform cutting endonuclease; cleaves Holliday junctions formed during recombination mitochondrial DNA
**Response to stress**				
SWI4	*CAGL0A04565g*	2185	3126	DNA binding component of the SBF complex (Swi4-Swi6)
BDF1	*CAGL0C02541g*	568	1932	Protein involved in transcription initiation; functions at TATA-containing promoters; associates with the basal transcription factor TFIID
**Filamentous growth**				
GLE1	*CAGL0J09636g*	756	1593	Cytoplasmic nucleoporin required for polyadenylated mRNA export; contains a nuclear export signal
**Protein catabolic process**				
YTA12	*CAGL0J01353g*	2343	2484	Mitochondrial inner membrane m-AAA protease component; mediates degradation of misfolded or unassembled proteins
**RNA metabolic process**				
MUD2	*CAGL0L05038g*	1159	1542	Protein involved in early pre-mRNA splicing; component of the pre-mRNA-U1 snRNP complex, the commitment complex
**Vesicle-mediated transport**				
CHC1	*CAGL0A03718g*	3608	4959	Clathrin heavy chain; subunit of the major coat protein involved in intracellular protein transport and endocytosis
**Mutants identified in the fluconazole sensitivity screen**				
**Lipid metabolic process**				
ERG4	*CAGL0A00429g*#	170	1395	C-24(28) sterol reductase; catalyzes the final step in ergosterol biosynthesis
UPC2	*CAGL0C01199g*#	838	2769	Sterol regulatory element binding protein; induces sterol biosynthetic genes
PDR16	*CAGL0J07436g*#	884	1035	Phosphatidylinositol transfer protein (PITP); controlled by the multiple drug resistance regulator Pdr1
FAB1	*CAGL0K10384g*	5681	6315	1-phosphatidylinositol-3-phosphate 5-kinase; vacuolar membrane kinase that generates phosphatidylinositol (3,5)P2, which is involved in vacuolar sorting and homeostasis
BST1	*CAGL0K12408g*	1751	3036	GPI inositol deacylase of the endoplasmic reticulum; negatively regulates COPII vesicle formation
**RNA metabolic process**				
PGD1	*CAGL0A01325g*#	1252	1434	Subunit of the RNA polymerase II mediator complex; associates with core polymerase subunits to form the RNA polymerase II holoenzyme
MED2	*CAGL0C04477g*#	318	1107	Subunit of the RNA polymerase II mediator complex; associates with core polymerase subunits to form the RNA polymerase II holoenzyme
HFI1	*CAGL0H00616g*	1113	1356	Adaptor protein required for structural integrity of the histone acetyltransferase-coactivator SAGA complex
SLT2	*CAGL0J00539g*#	557	1467	Serine/threonine MAP kinase of the PKC MAPK pathway
**Transport**				
PDR6	*CAGL0A00385g*	1503	3210	Karyopherin beta; responsible for import of the Toa1-Toa2 complex into the nucleus
ITR2	*CAGL0I07447g*	1446	1713	Myo-inositol transporter; member of the sugar transporter superfamily
THO2	*CAGL0J05654g*	2416	4707	Subunit of the THO complex; THO is required for efficient transcription elongation and involved in transcriptional elongation-associated recombination

FUN26	*CAGL0J08712g*	611	1242	High affinity, broad selectivity, nucleoside/nucleobase transporter; vacuolar membrane-localized transporter
**Cytoskeleton organization**				
BNR1	*CAGL0H06765g*#	1747	3885	Formin; nucleates the formation of linear actin filaments; involved in processes such as budding and mitotic spindle orientation which require the formation of polarized actin cables
BEM2	*CAGL0I06512g*#	1997	6360	Rho GTPase activating protein (RhoGAP); involved in the control of cytoskeleton organization and cellular morphogenesis; required for bud emergence
PAN1	*CAGL0J01892g*#	698	4125	Part of actin cytoskeleton-regulatory complex Pan1-Sla1-End3; associates with actin patches on cell cortex; promotes protein-protein interactions essential for endocytosis
**Regulation of nitrogen utilization**				
VID30	*CAGL0I04114g*	1174	2733	Central component of GID Complex, involved in FBPase degradation
URE2	*CAGL0J07392g*	224	1068	Nitrogen catabolite repression transcriptional regulator; inhibits GLN3 transcription in good nitrogen source
**Protein folding**				
EMC1	*CAGL0B00660g*	1459	2241	Member of conserved endoplasmic reticulum membrane complex; involved in efficient folding of proteins in the ER
**Biofilm formation**				
NRG2	*CAGL0L07480g*	630	945	Transcriptional repressor; mediates glucose repression and negatively regulates filamentous growth
**Miscellaneous**				
	CAGL0A00165g	16	840	
DNM1	*CAGL0D05808g*	8	2331	Dynamin-related GTPase involved in mitochondrial organization; required for mitochondrial fission and inheritance
EFR3	*CAGL0F03773g*	1637	2250	Protein required for Stt4-containing PI kinase complex localization
SAS2	*CAGL0K03773g*	793	1062	Histone acetyltransferase (HAT) catalytic subunit of the SAS complex; acetylates free histones and nucleosomes and regulates transcriptional silencing
NVJ2	*CAGL0K08030g*	657	2277	Lipid-binding ER protein, enriched at nucleus-vacuolar junctions
RPH1	*CAGL0L11880g*	1456	2943	JmjC domain-containing histone demethylase; targets tri- and dimethylated H3K36

Functional classification was performed using the GO Slim Mapper tool at CGD (http://www.candidagenome.org/cgi-bin/GO/goTermMapper).

#Genes previously implicated in azole tolerance.

Table S2: List of strains and plasmids used in the study.

Yeast strain	Genotype	References
BG2	Clinical isolate	Fidel *et al.*, 1996
YRK19	*ura3Δ::Tn903 G418R* (BG14)	Cormack and Falkow, 1999
YRK20	*URA3* (BG462)	Orta-Zavalza *et al.*, 2013
YRK531	*URA3 Cgbem2Δ::nat1*	Borah *et al.*, 2011
YRK695	*URA3 Cgvps15Δ::nat1*	Rai *et. al.*, 2015
YRK710	*URA3 Cgvps34Δ::nat1*	Rai *et. al.*, 2015
YRK870	*C. glabrata* isolate from the oral mucosa of a diabetic patient	Gift from Dr. Shukla Das
YRK981	*URA3 Cgprc1Δ::nat1*	Bairwa *et al.*, 2014
YRK1070	*ura3Δ::Tn903 G418R Cgfab1Δ::nat1*	This study
YRK1139	*ura3Δ::Tn903 G418R Cgfab1Δ::nat1/pRK1033*	This study
YPS1205	*URA3 Cgyps1-11Δ::hph*	Kaur *et. al.*, 2007
YRK1287	*URA3 Cgfab1Δ::nat1*	This study
YRK1297	*Cgpan1Δ::Tn7*	Borah *et al.*, 2011
YRK1413	*ura3Δ::Tn903 G418R* (YRK19)*/pRK1018*	This study
YRK1414	*ura3Δ::Tn903 G418R Cgfab1Δ::nat1/pRK1018*	This study
YRK1433	*ura3Δ::Tn903 G418R Cgfab1Δ::nat1/pRK1107*	This study
YRK1519	YRK20/*CgPDR1* ^{L280F}	This study
YRK1520	*ura3Δ::Tn903 G418R Cgfab1Δ/CgPDR1* ^{L280F}	This study
YRK1533	*ura3Δ::Tn903 G418R Cgfab1Δ::nat1/pRK1144*	This study
YRK1601	*URA3 Cgvac14Δ::nat1*	This study
YRK1692	*URA3 Cgvac7Δ::nat1*	This study
YRK1966	*Cgbnr1Δ::Tn7*	Borah *et al.*, 2011
YRK2144	*ura3Δ::Tn903 G418R* (YRK19)*/pRK1409*	This study
YRK2148	*ura3Δ::Tn903 G418R Cgfab1Δ::nat1/pRK1409*	This study
YRK2171	*ura3Δ::Tn903 G418R* (YRK19)*/pRK1411*	This study
YRK2181	*ura3Δ::Tn903 G418R Cgfab1Δ::nat1/pRK1411*	This study
YRK2359	*ura3Δ::Tn903 G418R* (YRK19)*/pRK1493*	This study
YRK2285	*ura3Δ::Tn903 G418R* (YRK19)*/pRK1476*	This study
YRK2287	*C. glabrata* isolate from the vitreous fluid of patient with ocular infection	Gift from Dr. S. Shivaji
YRK2288	*C. glabrata* isolate from the corneal scrapping of patient with ocular infection	Gift from Dr. S. Shivaji
YRK2289	*C. glabrata* isolate from the vitreous fluid of patient with ocular infection	Gift from Dr. S. Shivaji
YRK2290	*C. glabrata* isolate from the vitreous fluid of patient with ocular infection	Gift from Dr. S. Shivaji
YRK2291	*C. glabrata* isolate from the corneal scrapping of patient with ocular infection	Gift from Dr. S. Shivaji

YRK2301	*ura3Δ::Tn903 G418R Cgcap2Δ::nat1*	This study
YRK2315	*ura3Δ::Tn903 G418R Cgcap2Δ::nat1/pRK1409*	This study
YRK2340	*C. glabrata* isolate from the blood	Gift from Dr. Arunaloke Chakrabarti
YRK2341	*C. glabrata* isolate from the blood	Gift from Dr. Arunaloke Chakrabarti
YRK2342	*C. glabrata* isolate from the blood	Gift from Dr. Arunaloke Chakrabarti
YRK2344	*C. glabrata* isolate from the ascitic fluid	Gift from Dr. Arunaloke Chakrabarti
**Plasmid**	**Description**	**References**
pRK74	A CEN-ARS plasmid (pGRB2.2) of *C. glabrata* carrying *S. cerevisiae URA3* as a selection marker. MCS sites are flanked by *S. cerevisiae PGK1* promoter at one end and by 3′ UTR of *HIS3* at the other end.	Frieman *et al.*, 2002
pRK949	pSF67, plasmid with CgPDR1 hyperactive allele ($CgPDR1^{L280F}$) (DSY565)	Sanglard laboratory
pRK1018	pGRB2.3 plasmid	Addgene (Plasmid #45343)
pRK1033	*CgFAB1* (6.31 kb) cloned in BamH*I*-Sal*I* sites of pRK74 plasmid	This study
pRK1107	*CgFAB1* (6.31 kb) cloned in BamH*I*-Xma*I* sites of pGRB2.3 plasmid (pRK1018)	This study
pRK1144	$CgFAB1^{GKSG \rightarrow VKSV}$ cloned in the pRK74 plasmid	This study
pRK1349	SFB (S-protein-FLAG epitope-streptavidin-binding peptide) tag cloned in PCU-PDC1 (ADD Gene #45323) in *EcoRI-SalI* sites	Kaur Laboratory
pRK1409	*CgCAP2* (0.813 kb) cloned in Spe*I*-Xma*I* sites of pGRB2.3 plasmid (pRK1018)	This study
pRK1411	*CgCOF1* (0.908 kb) cloned in Spe*I*-BamH*I* sites of pGRB2.3 plasmid (pRK1018)	This study
pRK1476	*CgCOF1* (0.908 kb) cloned in Spe*I*-BamH*I* sites of pRK1349 plasmid	This study
pRK1493	*CgCAP2* (0.813 kb) cloned in Spe*I*-Xma*I* sites of pRK1349 plasmid	This study
pRK1494	*CgCOF1* cloned in pET28a vector at *EcoRI-XhoI* sites of pET28-a plasmid	This study

References

Bairwa, G., Rasheed, M., Taigwal, R., Sahoo, R., and Kaur, R. (2014) GPI (glycosylphosphatidylinositol)-linked aspartyl proteases regulate vacuole homoeostasis in *Candida glabrata*. *Biochem J* **458**: 323–334.

Borah, S., Shivarathri, R., and Kaur, R. (2011) The Rho1 GTPase-activating protein CgBem2 is required for survival of azole stress in *Candida glabrata*. *J Biol Chem* **286**: 34311–34324.

Cormack, B.P., and Falkow, S. (1999) Efficient homologous and illegitimate recombination in the opportunistic yeast pathogen *Candida glabrata*. *Genetics* **151**: 979–987.

Fidel, P.L., Cutright, J.L., Tait, L., and Sobel, J.D. (1996) A murine model of *Candida glabrata* vaginitis. *J Infect Dis* **173**: 425–431.

Frieman, M.B., McCaffery, J.M., and Cormack, B.P. (2002) Modular domain structure in the *Candida glabrata* adhesin Epa1p, a beta1,6 glucan-cross-linked cell wall protein. *Mol Microbiol* **46**: 479–492.

Kaur, R., Ma, B., and Cormack BP (2007) A family of glycosylphosphatidylinositol-linked aspartyl proteases is required for virulence of *Candida glabrata*. *Proc Natl Acad Sci USA* **104:** 7628-7633

Orta-Zavalza, E., Guerrero-Serrano, G., Gutierrez-Escobedo, G., Canas-Villamar, I., Juarez-Cepeda, J., Castano, I., and Las Penas, A. De (2013) Local silencing controls the oxidative stress response and the multidrug resistance in *Candida glabrata*. *Mol Microbiol* **88**: 1135–1148.

Rai, M. N., Sharma, V., Balusu, S., and Kaur, R. 2015. An essential role for phosphatidylinositol 3-kinase in the inhibition of phagosomal maturation, intracellular survival and virulence in *Candida glabrata*. *Cell Microbiol* **17:** 269-287.

Table S3: List of primers used in the study.

Primer	Sequence (5'→ 3')	Description
**For generation of deletion strains**		
OgRK986	CGTCCGCAAAATCTTCATCT	*CgFAB1* 5'UTR Forward
OgRK987	GCGTCGACCTGCAGCGTACGATAGGCCAACGCAACTCATT	*CgFAB1* 5'UTR Reverse
OgRK988	CGACGGTGTCGGTCTCGTAGCACCAACTGCCGTGTAAGGT	*CgFAB1* 3'UTR Forward
OgRK989	ATGACAGAAATAGCGCAGCA	*CgFAB1* 3'UTR Reverse
OgRK990	GAGTCATCGTCGCTCCATTC	*CgFAB1* 5' Integration check Forward
OgRK991	TATTACCGCCTGCAACTCCT	*CgFAB1* 3' Integration check Reverse
OgRK1366	CTGGAACGGATAAGCGAGAG	*CgFAB1* Internal check Forward
OgRK1367	TCAACTGCGTCCTTGACATC	*CgFAB1* Internal check Reverse
OgRK1954	GGATTGGATCAGCAAACCACTTA	*CgPDR1* 5' integration check
OgRK1955	ACAACCATGAAGACAGACAAAGA	*CgPDR1* 3' integration check
OgRK2746	CTTGGAAAGAGTGGGTTCCAGAG	*CgCAP2* 5'UTR Forward
OgRK2747	GCGTCGACCTGCAGCGTACGCGTAAGGGAAGAAGCTCTGTG	*CgCAP2* 5'UTR Reverse
OgRK2748	CGACGGTGTCGGTCTCGTAGCCCACCAACAAAATGAAACTC	*CgCAP2* 3'UTR Forward
OgRK2749	CTCGGACCAAACTCTGCATCTC	*CgCAP2* 3'UTR Reverse
OgRK2750	GCACTACTGAACGACTTGCCAC	*CgCAP2* 5' Integration check Forward
OgRK2751	CTCGATCTCATAATCTTTGAC	*CgCAP2* 3' Integration check Reverse
OGRK 2653	TGCTGCACTTGATCTGCTTC	*CgCAP2* Internal check Forward
OGRK 2654	ATGGTGATTCCTGCAGATCC	*CgCAP2* Internal check Reverse
OgRK2123	CAGCACAGACTCCAACTTCTCCCTG	*CgVAC7* 5'UTR Forward
OgRK2124	GCGTCGACCTGCAGCGTACGCAACTTCTACCTTAGTGTGG	*CgVAC7* 5'UTR Reverse
OgRK2125	CGACGGTGTCGGTCTCGTAGCGGTGAACTGCATTCCACC	*CgVAC7* 3'UTR Forward
OgRK2126	CCTCGATACTTCACTAGACTAAGAG	*CgVAC7* 3'UTR Reverse
OgRK2127	CAGAGGACAGCAAAGGCTCAAC	*CgVAC7* Internal check Forward
OgRK2128	GAGCCCACTTCATTGTGTTCCG	*CgVAC7* Internal check Reverse
OgRK2129	GACCACTCACACCACCATACAG	*CgVAC7* 5' Integration check Forward
OgRK2130	CGTTGTTATAAATATAGTCGGTAC	*CgVAC7* 3' Integration check Reverse
OgRK2131	CCAGGACACTGACAAAAGGACCC	*CgVAC14* 5'UTR Forward
OgRK2132	GCGTCGACCTGCAGCGTACGGCTGATCCTGATCCAAGGTG	*CgVAC14* 5'UTR Reverse
OgRK2133	CGACGGTGTCGGTCTCGTAGCACATGAATAGGATTATGAG	*CgVAC14* 3'UTR Forward
OgRK2134	GCACATATGGACTCCAAGCGTC	*CgVAC14* 3'UTR Reverse
OgRK2135	CTTATGACGGCTCCTGAGCTT	*CgVAC14* Internal check Forward
OgRK2136	GTTGAACGCTTCAGACTGTGG	*CgVAC14* Internal check Reverse

OgRK2137	GTATGCCACATCGGGAGAAAC	*CgVAC14* 5' Integration check Forward
OgRK2138	GTGTTGCAATTATCAAGGCTCC	*CgVAC14* 3' Integration check Reverse
**For gene cloning**		
OgRK1177	CGCGGATCCATGGCTATTGGTCAAGGGACG	*CgFAB1* Cloning Forward
OgRK1178	CGCGTCGACTTAGTTTGTATCTTGATACCA	*CgFAB1* Cloning Reverse, SDM 2nd half Reverse
OgRK1903	CCGTGGATCCATGGCTATTGGTCAAGGGACGC	*CgFAB1* Cloning Forward
OgRK1904	CGATCCCGGGGTTTGTATCTTGATACCATGGG	*CgFAB1* Cloning Reverse
OgRK2628	CAGAACTAGTATGGATGAGAAATATGATGC	*CgCAP2* Cloning Forward
OgRK2629	CTGTCCCGGGTAGATTTTGAAGACCCTTGAT	*CgCAP2* Cloning Reverse
OgRK2630	CAGAACTAGTATGTCCAGATCAGGGTATGT	*CgCOF1* Cloning Forward
OgRK2631	GTCTGGATCCGTGAGAACCAGCACCTCTGC	*CgCOF1* Cloning Reverse
OgRK2739	GTAGAATTCGATTACAAGGATGACGACGATAAGATGTCCAGA TCAGGTGTTGCCG	*CgCOF1* Cloning in pET28a Forward
OgRK2740	GTACTCGAGTTAGTGAGAACCAGCACCTCTGC	*CgCOF1* Cloning in pET28a Reverse
**For qRT-PCR**		
OgRK127	TGCAGGACCAAGTCAGACAG	*CgCDR1* Forward
OgRK128	CTCATCGGAAGTAGGGTCCA	*CgCDR1* Reverse
OGRK133	ACGGTACCAAGCCATACGAG	*CgERG11* Forward
OGRK134	GAACACTGGGGTGGTCAAGT	*CgERG11* Reverse
OgRK135	AAAGGGAGTGACAGCGAGAA	*CgPDR1* Forward
OgRK136	CTCAATGGCGTCAATGGATGA	*CgPDR1* Reverse
OgRK191	TTTCAGAGTGCCAACTGTCG	*CgGAPDH* Forward
OgRK192	TGAAACAACAGCGTCCTCAG	*CgGAPDH* Reverse
OGRK1535	GACCGTGAGAAGGTCTTGGA	*CgERG1* Forward
OGRK1536	TCACCACCACGTTGGAAATA	*CgERG1* Reverse
OGRK1537	TCGACGGTTACTTCCAATCC	*CgERG3* Forward
OGRK1538	TTGACAACTGGGTTGTTGGA	*CgERG3* Reverse
OGRK1539	CCCATTTTTGCCATACCAAG	*CgERG4* Forward
OGRK1540	AACCACAAGACATAGCCCAGA	*CgERG4* Reverse
OGRK1541	AAGGATCTTGCTGACGAGGA	*CgERG6* Forward
OGRK1542	CGACACCAACCTTTTCCATT	*CgERG6* Reverse
OGRK2015	CAGGATTCTAGTGATGAAGAG	*CgFAB1* Forward
OGRK2016	TGGAGGTGGAGGAGGAGTGGA	*CgFAB1* Reverse
**For site-directed mutagenesis**		
OgRK2025	GTCGTCCGATTAGAATATTCTG	*CgFAB1* SDM 2nd half Forward
OgRK2030	GTGGCATTCAAACGGTGTAAAATCTGTGAGTGGATTCTTAAAG AC	*CgFAB1* SDM 1st half Forward
OgRK2031	GTCTTTAAGAATCCACTCACAGATTTTACACCGTTTGAATGCC AC	*CgFAB1* SDM 1st half Reverse

Figure S1

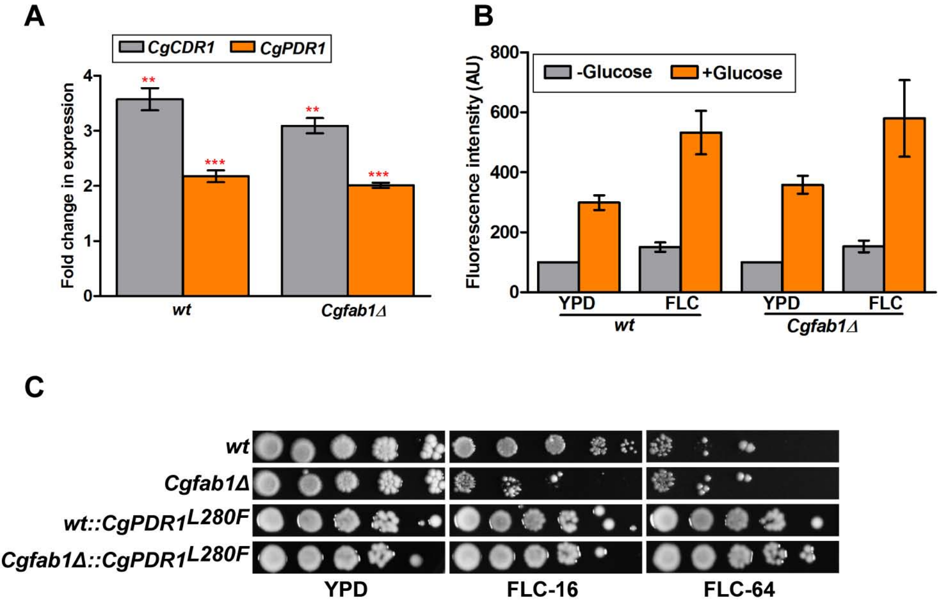
A
CgCDR1
CgPDR1
Fold change in expression
0
1
2
3
4
**
***
**
***
wt
Cgfab1Δ
B
-Glucose
+Glucose
Fluorescence intensity (AU)
0
200
400
600
800
YPD
FLC
YPD
FLC
wt
Cgfab1Δ
C
wt
Cgfab1Δ
wt::CgPDR1L280F
Cgfab1Δ::CgPDR1L280F
YPD
FLC-16
FLC-64

Figure S2

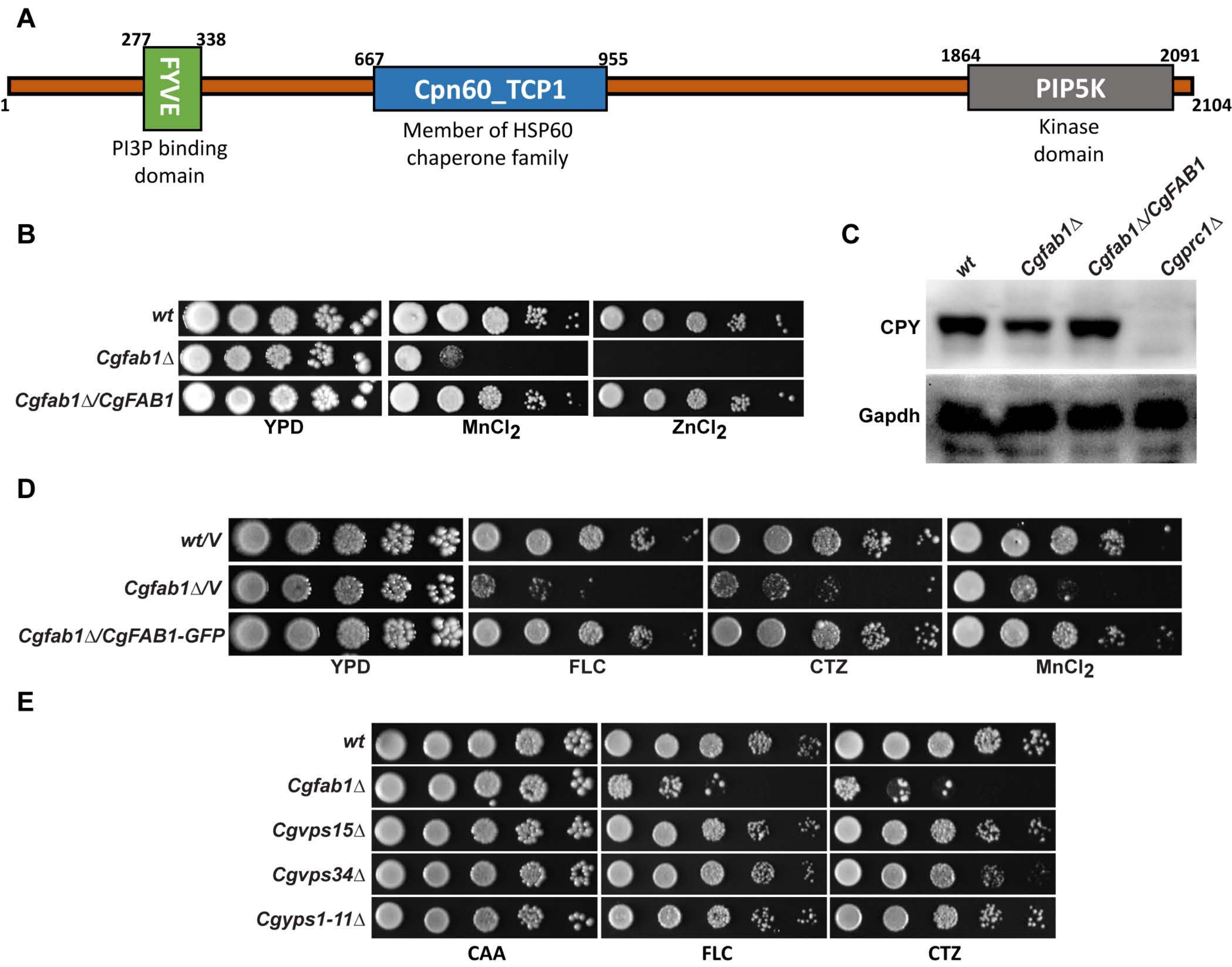
A
277
338
FYVE
PI3P binding domain
667
955
Cpn60_TCP1
Member of HSP60 chaperone family
1864
2091
PIP5K
Kinase domain
1
2104
B
wt
Cgfab1Δ
Cgfab1Δ/CgFAB1
YPD
MnCl2
ZnCl2
C
wt
Cgfab1Δ
Cgfab1Δ/CgFAB1
Cgprc1Δ
CPY
Gapdh
D
wt/V
Cgfab1Δ/V
Cgfab1Δ/CgFAB1-GFP
YPD
FLC
CTZ
MnCl2
E
wt
Cgfab1Δ
Cgvps15Δ
Cgvps34Δ
Cgyps1-11Δ
CAA
FLC
CTZ

Figure S3

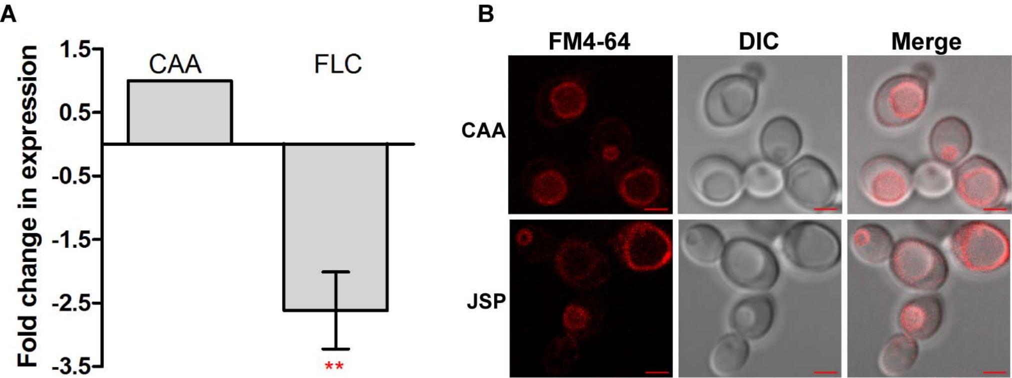
A
1.5
0.5
-0.5
-1.5
-2.5
-3.5
Fold change in expression
CAA
FLC
**
B
FM4-64
DIC
Merge
CAA
JSP

Figure S4

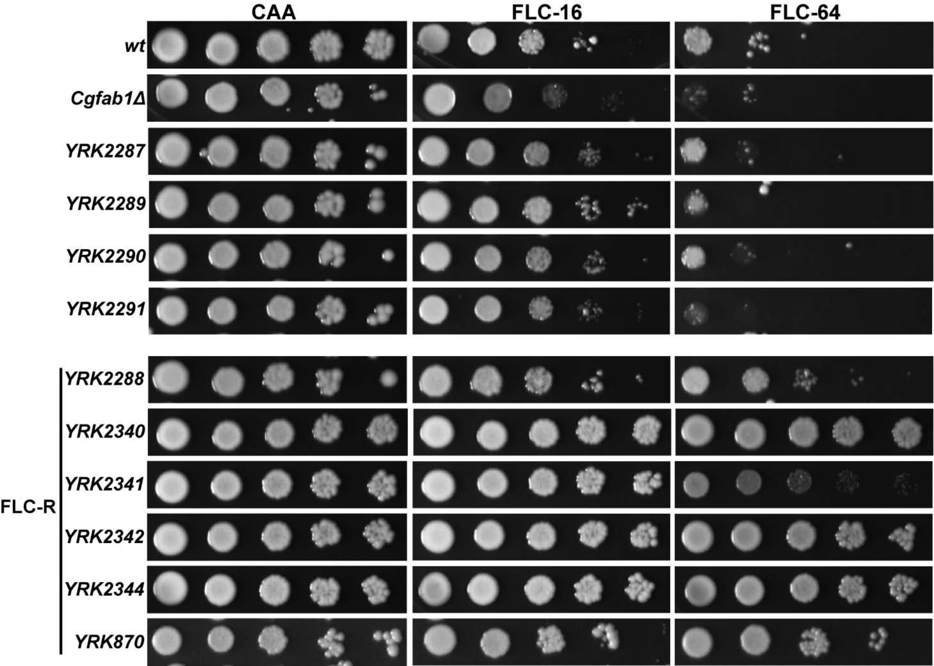
CAA
FLC-16
FLC-64
wt
Cgfab1Δ
YRK2287
YRK2289
YRK2290
YRK2291
YRK2288
YRK2340
YRK2341
YRK2342
YRK2344
YRK870
FLC-R

Figure S5

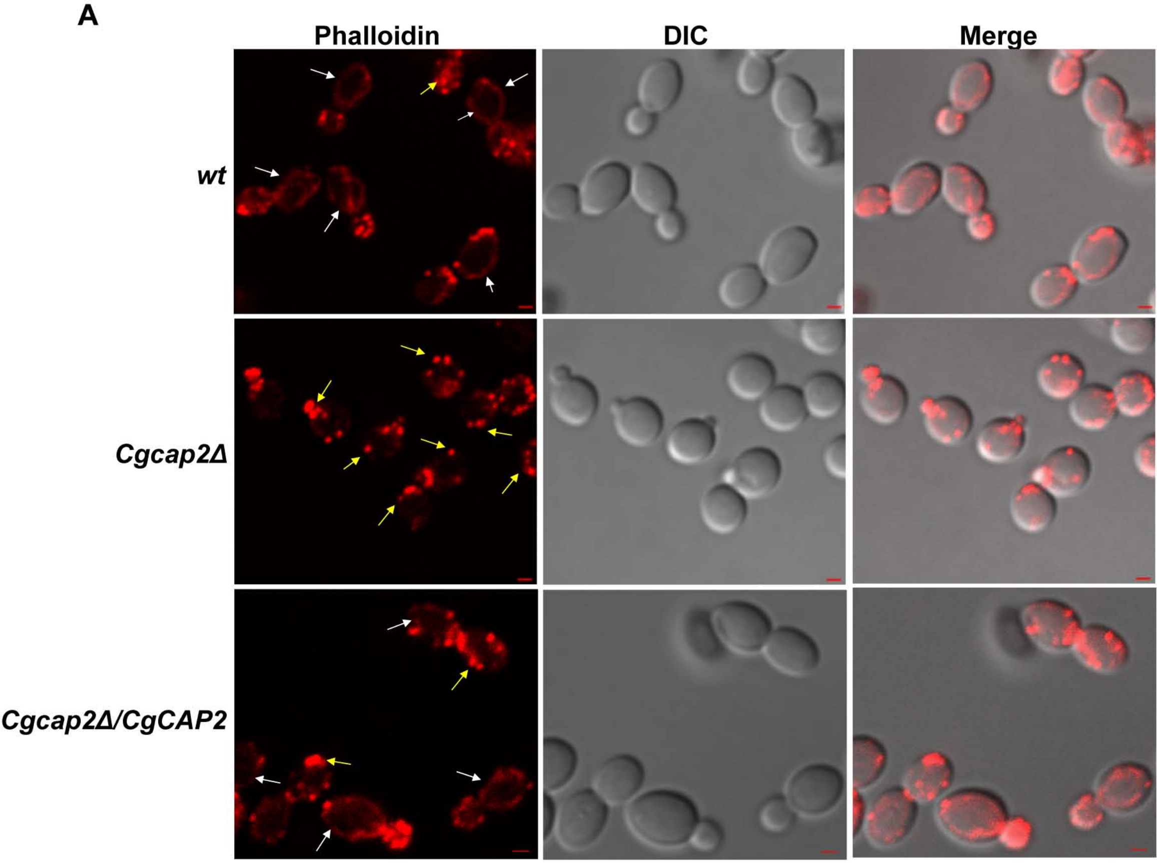
A
Phalloidin
DIC
Merge
wt
Cgcap2Δ
Cgcap2Δ/CgCAP2

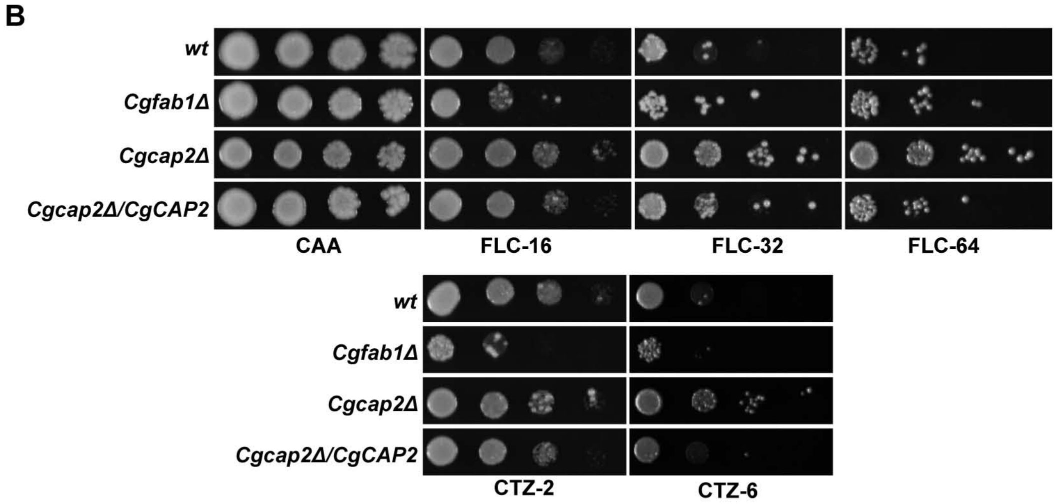
B
wt
Cgfab1Δ
Cgcap2Δ
Cgcap2Δ/CgCAP2
CAA
FLC-16
FLC-32
FLC-64
wt
Cgfab1Δ
Cgcap2Δ
Cgcap2Δ/CgCAP2
CTZ-2
CTZ-6

Figure S6

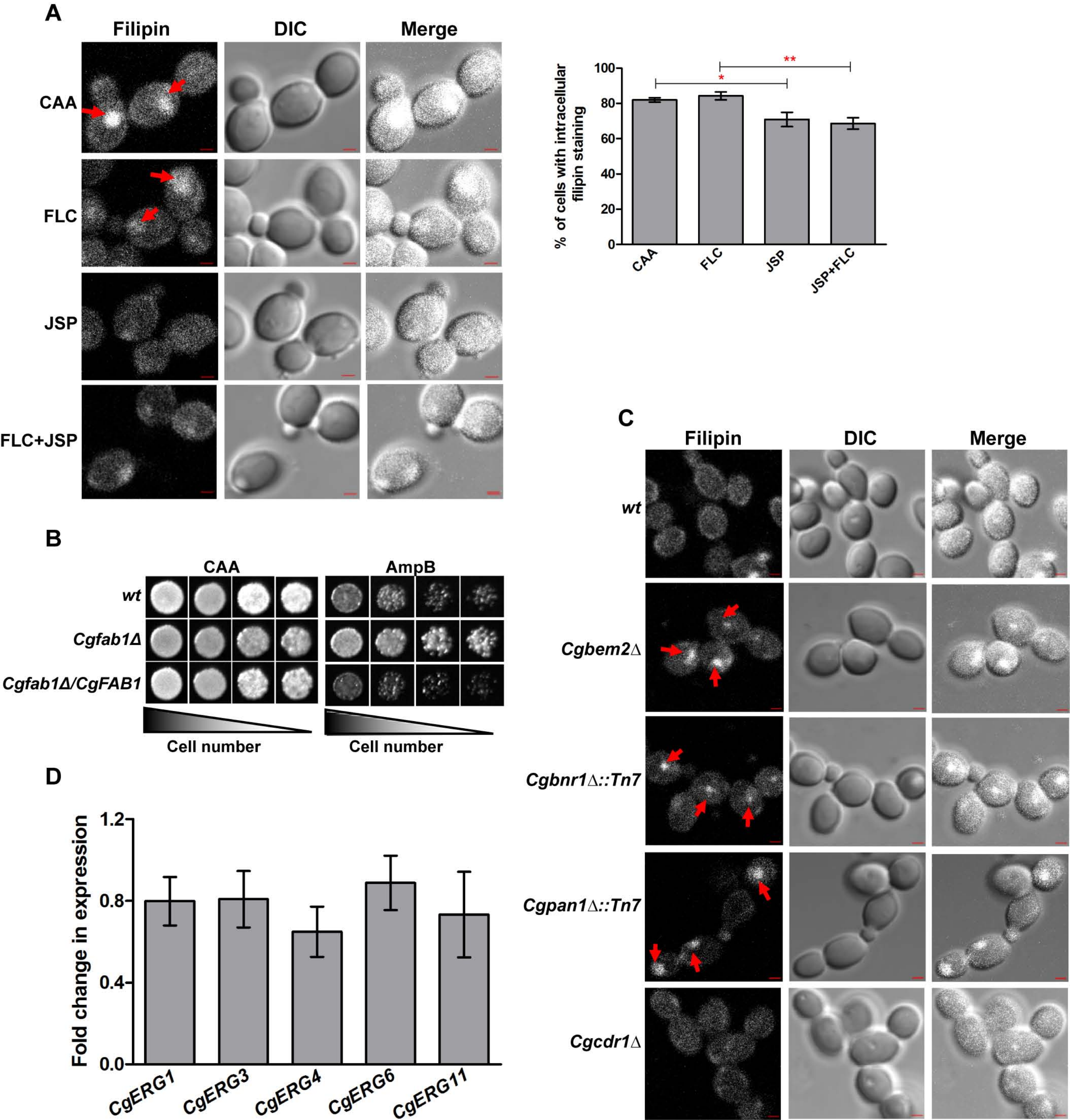

A
Filipin
DIC
Merge
CAA
FLC
JSP
FLC+JSP
% of cells with intracellular filipin staining
100
80
60
40
20
0
CAA
FLC
JSP
JSP+FLC
*
**
B
CAA
AmpB
wt
Cgfab1Δ
Cgfab1Δ/CgFAB1
Cell number
Cell number
C
Filipin
DIC
Merge
wt
Cgbem2Δ
Cgbnr1Δ::Tn7
Cgpan1Δ::Tn7
Cgcdr1Δ
D
Fold change in expression
1.2
0.8
0.4
0.0
CgERG1
CgERG3
CgERG4
CgERG6
CgERG11

Figure S7

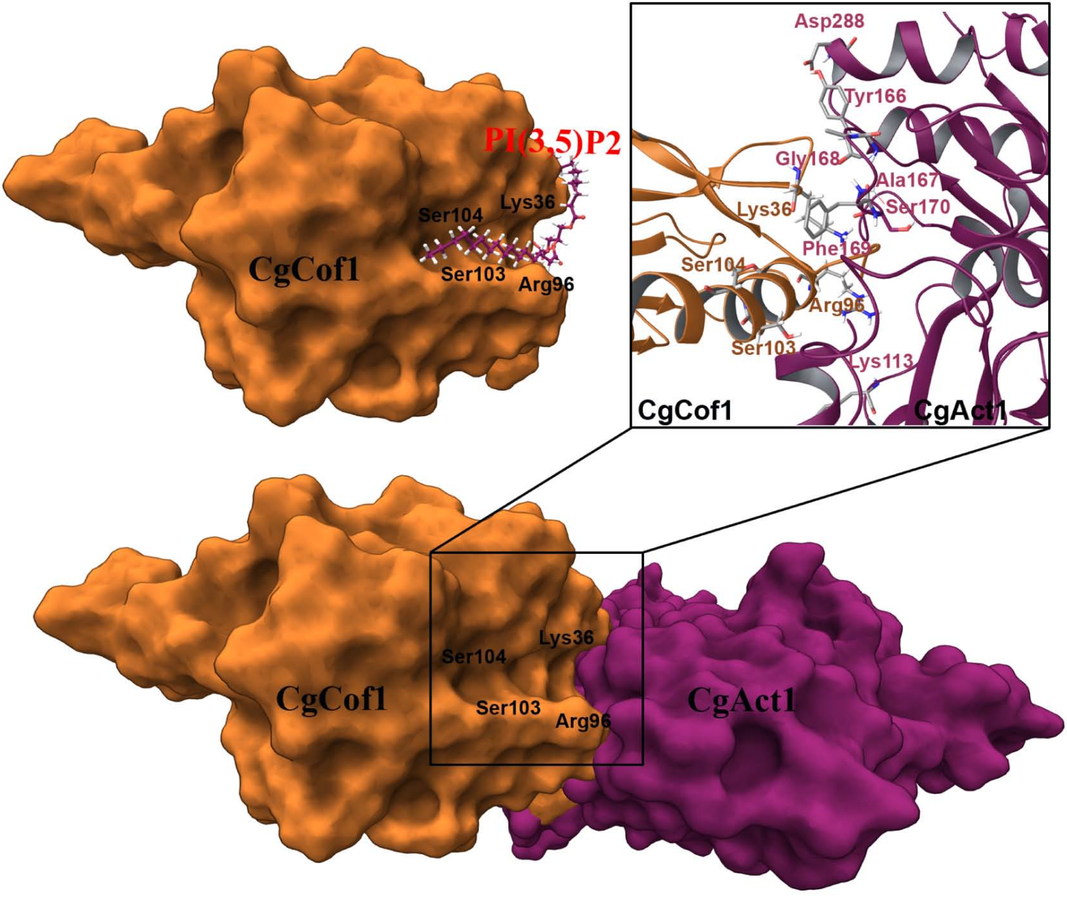
PI(3,5)P2
Lys36
Ser104
CgCof1
Ser103
Arg96
Asp288
Tyr166
Gly168
Ala167
Lys36
Ser170
Phe169
Ser104
Arg96
Ser103
Lys113
CgCof1
CgAct1
Lys36
Ser104
CgCof1
Ser103
Arg96
CgAct1

Figure S8

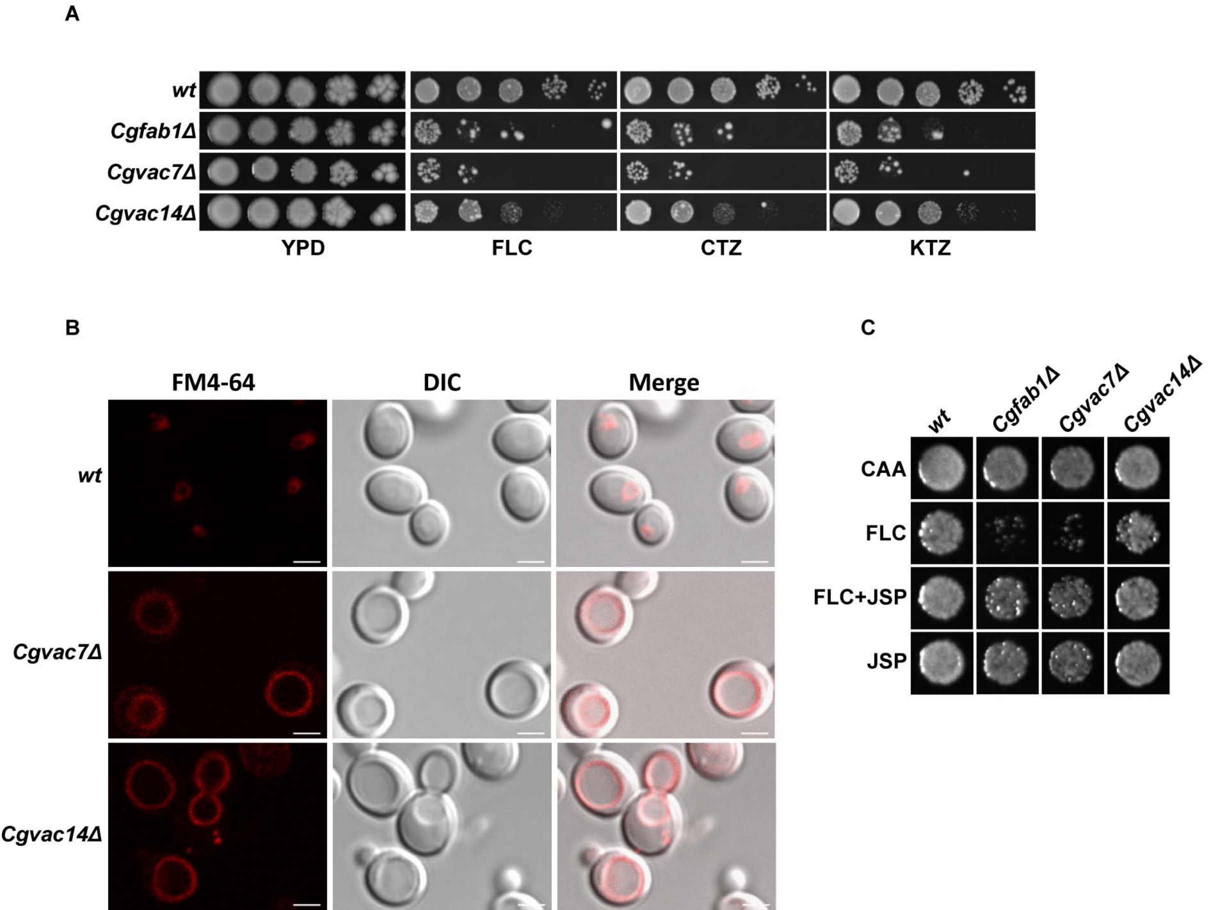
A
wt
Cgfab1Δ
Cgvac7Δ
Cgvac14Δ
YPD
FLC
CTZ
KTZ
B
FM4-64
DIC
Merge
wt
Cgvac7Δ
Cgvac14Δ
C
wt
Cgfab1Δ
Cgvac7Δ
Cgvac14Δ
CAA
FLC
FLC+JSP
JSP
